# Unraveling the Effects of Depolarization in Ultrathin Barium Titanate

**DOI:** 10.1002/advs.78103

**Published:** 2026-10-31

**Authors:** Thomas Lee, Prabhat Kumar, Christoph Klewe, Pravin Kavle, Padraic Shafer, Zhi (Jackie) Yao, Lane W. Martin

**Affiliations:** ^1^ Department of Materials Science and Engineering University of California Berkeley California USA; ^2^ Materials Sciences Division Lawrence Berkeley National Laboratory Berkeley California USA; ^3^ Rice Advanced Materials Institute Rice University Houston Texas USA; ^4^ Advanced Light Source Lawrence Berkeley National Laboratory Berkeley California USA; ^5^ National Synchrotron Light Source II Brookhaven National Laboratory Upton New York USA; ^6^ Applied Mathematics and Computational Research Division Lawrence Berkeley National Laboratory Berkeley California USA; ^7^ Departments of Materials Science and NanoEngineering Chemistry, and Physics and Astronomy Rice University Houston Texas USA

**Keywords:** ${\mathrm{BaTiO}}_{3}$, beyond‐CMOS, depolarization field, domain switching, ferroelectric thin films

## Abstract

Depolarization fields pose a critical obstacle to realizing ultrathin ferroelectric devices with low‐voltage operation. Although the phenomenology of depolarization has been observed for decades, whether polarization loss proceeds through suppression of ferroelectric order or reverse‐domain formation has remained unresolved. Here, a combination of experiment and simulation resolves this question and establishes the electrostatic origin of polarization instability in the ultrathin limit for BaTiO_3_. Synchrotron X‐ray absorption spectroscopy confirms that the polar structural distortion in ${\mathrm{BaTiO}}_{3}$ persists down to (at least) 3 nm even as the remanent polarization falls sharply, identifying reverse‐domain formation as the primary mechanism of polarization loss. Time‐resolved pulsed‐switching measurements reveal dynamically evolving depolarization fields that converge to a thickness‐independent equilibrium value. An electrostatic model attributes these fields to the effective series capacitance of the electrode interfaces, validated through thickness scaling and dielectric‐buffer experiments. Phase‐field simulations reveal that modifying this series capacitance reshapes switching dynamics by redistributing internal fields without altering the intrinsic ferroelectric energy landscape. Defects play a consequential secondary role, independently controlling relaxation pathways within this electrostatically constrained regime. These results establish depolarization as a system‐level effect governed by heterostructure boundary conditions, and identify interfacial design and defect management as complementary strategies for stabilizing polarization in ultrathin ferroelectrics.

## Graphical abstract

This article employs synchrotron‐based spectroscopy, time‐resolved electrical measurements, and phase‐field simulations to establish reverse‐domain formation as the mechanism of polarization loss in ultrathin ${\mathrm{BaTiO}}_{3}$ films as a critical obstacle to low‐voltage ferroelectric devices. The work develops a system‐level framework in which electrode screening and interfacial capacitance are found to drive depolarization and explores interfacial design and defect engineering as potential routes to tune polarization stability.

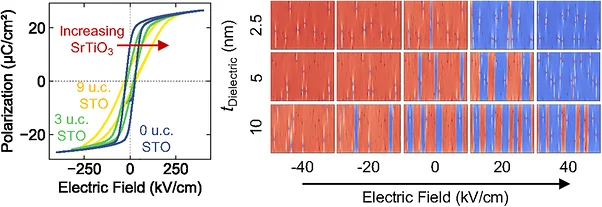

## 1 Introduction

Ferroelectrics, by virtue of their switchable polarization, are increasingly being explored for beyond‐complementary metal‐oxide‐semiconductor (CMOS) computing and data‐storage architectures [1, 2, 3]. Among these materials, ${\mathrm{BaTiO}}_{3}$ is notable for its exceptionally low coercive field (<20 kV ${\mathrm{cm}}^{-1}$) when realized as high‐quality thin films, with recent work demonstrating sub‐100 mV switching in ${\mathrm{BaTiO}}_{3}$ capacitors thinner than 50 nm [4]. In contrast to commercial ferroelectric‐based logic and memory devices, which typically operate at voltages exceeding 0.5–1 V [5, 6], ${\mathrm{BaTiO}}_{3}$‐based devices present a pathway to lower the energy of computation as compared with CMOS operating at ≈ 0.7 V [7].

Scaling to the ultrathin regime necessary for such low‐voltage operation, however, is (generally) accompanied by a pronounced reduction in remanent polarization, commonly attributed to enhanced depolarization fields at reduced thickness. While depolarization degrades polarization stability–posing challenges for non‐volatile functionality – it can simultaneously drive favorable deviation from conventional Janovec–Kay–Dunn‐type coercive‐field scaling [8, 9], thereby enabling the low‐field switching observed in ultrathin ${\mathrm{BaTiO}}_{3}$ [4, 10]. Deliberately enhanced, depolarization fields have likewise been shown to accelerate domain nucleation and reduce imprint [11]. This dual role establishes depolarization as both a limitation and a functional enabler, highlighting the need for a clear physical understanding of the mechanisms giving rise to device‐level depolarization in ultrathin ferroelectrics.

Depolarization has been examined for decades through theoretical and experimental approaches, yet fundamental questions remain unresolved. Early work emphasized the role of charge compensation at interfaces and electrode screening in enabling polarization retention [12, 13]. Subsequent studies connected depolarization to interfacial polarization gradients, dead layers, and Thomas‐Fermi screening lengths in electrodes [10, 14, 15, 16, 17], while recent efforts have identified external‐circuit capacitance as an additional contributor [18]. Direct observations of depolarization‐driven 180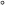 domain formation, in particular, typically concern the as‐grown and equilibrium states of ultrathin films – demonstrated where the ferroelectric is electrostatically isolated from its metallic screening interfaces by inserted dielectric layers [11, 19], transiently during electrode growth while the nascent electrode remains too thin to screen effectively [20], and near the morphotropic phase boundary of ${\mathrm{PbZr}}_{0.52}{\mathrm{Ti}}_{0.48}O_{3}$, where a flattened energy landscape stabilizes continuously variable populations of oppositely poled nanodomains [21] – while reductions in tetragonality near the critical thickness point instead toward suppression of the polar distortion itself [22], particularly via strong interfacial gradients [23, 24]. A working capacitor, by contrast, loses its remanent polarization at thicknesses well above the critical limit, with fully metallic electrodes in place – and further loses it dynamically, relaxing from a switched state rather than the equilibrium of an as‐grown one. Which mechanism governs that loss, and in what proportion, has thus remained an open question: the structural observations and the electrical measurements have, to date, largely been made on different systems, with differing boundary conditions, in different thickness regimes.

Therefore, the present investigation seeks to: (1) determine whether polarization instability in ultrathin ${\mathrm{BaTiO}}_{3}$ reflects suppression of ferroelectric order or arises from depolarization‐driven domain formation, (2) identify the physical origin and magnitude of the depolarization fields responsible for this instability, and (3) establish a framework for quantitatively predicting polarization stability under realistic device conditions. A key hypothesis underlying this work is that depolarization, rather than reflecting an intrinsic material limitation, is fundamentally a system‐level phenomenon governed by the electrostatic environment in which the ferroelectric is embedded. If this view is correct, then the primary degree of freedom for controlling polarization stability lies not in the ferroelectric material itself but in how the electrostatic boundary conditions dictate the driving force for instability, while material energetics and defect landscapes govern the relaxation pathways through domain nucleation, growth, and pinning.

The instability of polarization in ultrathin ${\mathrm{BaTiO}}_{3}$ is found to be mediated by the formation of depolarization‐driven reverse domains, via internal fields that evolve dynamically during relaxation as a consequence of incomplete screening in nanoscale heterostructures. This work clarifies how polarization stability is controlled through electrostatic boundary conditions and demonstrates that this stability can be engineered through interfacial design and defect management – providing a foundation for stabilizing ultrathin ferroelectrics designed for low‐voltage operation.

## 2 Results and Discussion

### 2.1 Reverse‐Domain Formation as the Mechanism of Polarization Instability

Here, ${\mathrm{BaTiO}}_{3}$ is employed as a model system for studying depolarization since, under compressive epitaxial strain, it adopts a well‐defined tetragonal phase with simple 180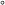 domain walls, allowing the effects of film geometry and electrostatic boundary conditions to be studied without the complication of concurrent phase transitions or complex domain‐structure evolution. Symmetric heterostructures consisting of 30 nm ${\mathrm{SrRuO}}_{3}$/25–100 nm ${\mathrm{BaTiO}}_{3}$/30 nm ${\mathrm{SrRuO}}_{3}$ and asymmetric heterostructures of 3–100 nm ${\mathrm{BaTiO}}_{3}$/30 nm ${\mathrm{SrRuO}}_{3}$ were grown on ${\mathrm{GdScO}}_{3}$ (110) substrates via pulsed‐laser deposition (Experimental Section) to study the thickness evolution of depolarization effects. Following growth, X‐ray diffraction ω–2θ line scans (Figure 1a) and reciprocal space maps (Figure S1) confirm all films are single‐phase, fully epitaxial, and coherently strained to the substrate. Polarization‐electric field hysteresis‐loop measurements (Experimental Section) reveal a clear reduction in remanent polarization and an increase in loop slant with decreasing ${\mathrm{BaTiO}}_{3}$ thickness, while the coercive field remains largely unchanged (Figure 1b).

**FIGURE 1:**
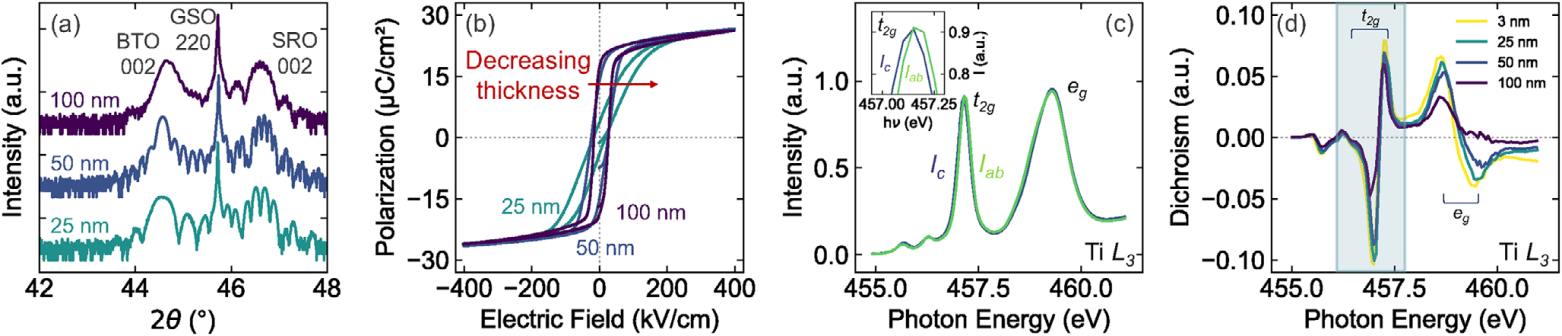
Structural characterization and polarization behavior of ultrathin ${\mathrm{BaTiO}}_{3}$. (a) ω–2θ scans showing fully epitaxial ${\mathrm{BaTiO}}_{3}$ films from 25 to 100 nm thick. (b) Polarization‐electric field hysteresis loops demonstrating reduced remanent polarization ($P_{r}$) and increased slant as the ${\mathrm{BaTiO}}_{3}$ thickness decreases. (c) Representative XAS spectra at the titanium $L_{3}$ edge from a 50‐nm‐thick ${\mathrm{BaTiO}}_{3}\mathrm{film}$, with the inset showing the subtle splitting of the $t_{2g}$ peak. (d) Titanium $L_{3}$‐edge X‐ray linear dichroism signal revealing persistent features indicative of robust polar distortion across all thicknesses studied herein.

Although the polarization‐electric field hysteresis‐loop measurements reveal a reduction in polarization stability with reduced ${\mathrm{BaTiO}}_{3}$ thickness, they are insufficient to determine whether this arises from a suppression of the polar distortion or the formation of reverse domains. To directly assess the polar distortion, synchrotron‐based X‐ray absorption spectroscopy (XAS) and X‐ray linear dichroism (XLD) measurements were conducted on uncapped ${\mathrm{BaTiO}}_{3}$ films ranging from 100 nm thick (where polarization is known to be robust) to just 3 nm thick (below the limit of what is feasible for typical capacitor‐based measurements) (Experimental Section). The ferroelectric polarization in ${\mathrm{BaTiO}}_{3}$ originates from a structural distortion associated with the titanium off‐centering within the oxygen octahedra. This off‐centering lowers the local‐site symmetry and lifts the degeneracy of the titanium 3d levels, so that X‐rays polarized along and perpendicular to the polar axis probe orbitals of different energy; the resulting dichroism at the $t_{2g}$ feature, and its sign, constitute the spectroscopic signature of the polar state [25, 26] (Supporting Information). Critically, this measurement probes the local structural (tetragonal) distortion of individual unit cells independently of the macroscopic domain configuration, and so tests whether the polar distortion is suppressed, regardless of the domain configuration. It also reports the symmetry of the distortion and its relative magnitude between samples rather than an absolute dipole moment, so while a reduction in dipole amplitude uniform to the series would not be resolved, variation with sample thickness would be detectable. The tetragonality it reflects likewise carries non‐polar contributions, such as those of epitaxial strain and, in principle, point defects. Because the films are grown identically and the coherently strained samples share essentially the same strain state, such contributions are expected to be common across the series rather than sample specific, so it is the evolution of the dichroism across the series – rather than its magnitude in any one film – that speaks to the polar distortion. Grazing‐incidence XAS/XLD spectra at the titanium L edge collected using in‐plane and out‐of‐plane polarization (Figure 1c) show a dichroism at the titanium $L_{3}$ edge that persists, with unchanged sign, across all thicknesses down to 3 nm (Figure 1d). Its amplitude is essentially unchanged from 3 to 50 nm; only the 100‐nm‐thick films, which are known to be robustly polar, show a reduced amplitude corresponding to the onset of partial relaxation evident in the reciprocal space maps (Figure S1), reflecting the strain state of the near‐surface region sampled by the measurement rather than the polarization. The polar distortion (and, by extension, ferroelectricity) is therefore maintained even in films just 3 nm thick – near the predicted critical thickness for ferroelectricity in ${\mathrm{BaTiO}}_{3}$ and below the thicknesses accessible to direct polarization measurement due to leakage constraints. The polar signature further shows no systematic weakening as the thickness is reduced, even as the remanent polarization falls sharply across the same range, indicating that the substantial reduction of macroscopic remanent polarization ($P_{r}$) at low thickness arises predominantly from reverse‐domain formation rather than suppression of the polar distortion.

### 2.2 Dynamic Evolution of Depolarization Fields

With reverse‐domain formation established as the mechanism of polarization reduction, attention turns to the magnitude and temporal evolution of the depolarization fields driving this process. Although ferroelectric switching kinetics have been extensively studied [27] – ranging from Merz's early description of field‐activated domain‐wall motion [28, 29] to more recent treatments incorporating nucleation and growth [30, 31], impingement [32], and dimensionality effects [33] – these frameworks have been applied overwhelmingly to forward switching under applied electric fields. Polarization relaxation following forward switching, by contrast, proceeds under an internally generated depolarization field whose magnitude and temporal evolution are not directly accessible from static‐hysteresis measurements.

A positive‐up‐negative‐down (PUND)‐style measurement, however, can provide access to these dynamics, capturing polarization stability over timescales from 30 ns to 800 μs using sequential switching pulses (Experimental Section). This approach yields a pair of switching transients (Figure 2a). The first pulse sets the fully poled state, and the delayed second pulse probes the remanent polarization after a given relaxation period. Integration of the differential between the transient responses provides the transient remanent polarization as a function of delay time. Larger areas under the second pulse correspond to greater polarization loss with increasing delay. Polarization retention curves (Figure 2b) derived from the pulsed‐switching data show a further decrease in remanent polarization with decreasing ${\mathrm{BaTiO}}_{3}$ thickness. All films exhibit power‐law behavior at long times – behavior typically associated with domain‐wall creep due to thermal fluctuations [18]. Additionally, that the relaxation unfolds over tens of nanoseconds to hundreds of microseconds, rather than the picosecond timescales expected of a soft‐mode collapse of the polar order, is further evidence of relaxation being mediated primarily by the nucleation and lateral growth of reverse domains.

**FIGURE 2:**
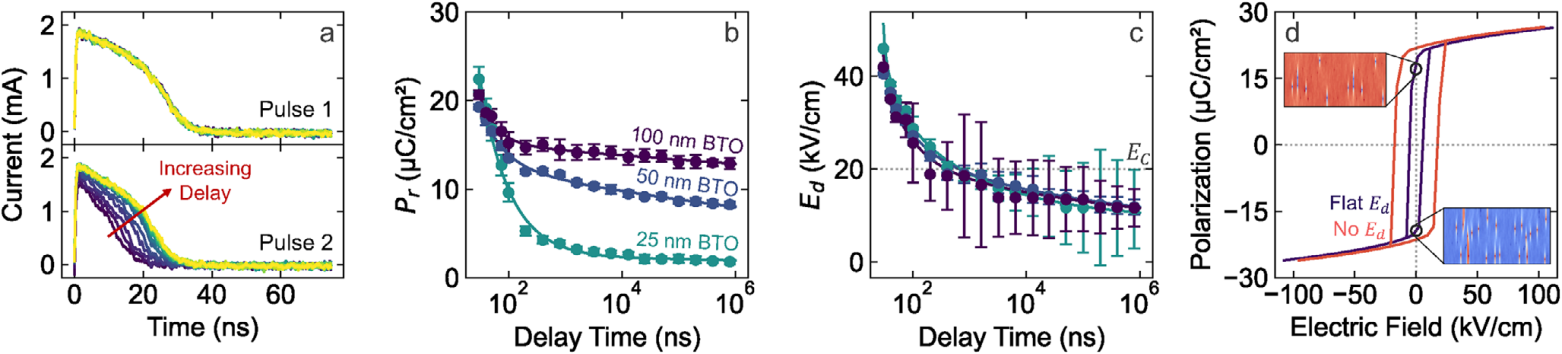
Time‐resolved depolarization‐field dynamics across the ${\mathrm{BaTiO}}_{3}$ thickness series. (a) PUND data showing increased switching currents in the second pulse as remanent polarization decays with delay time. (b) Extracted transient remanent polarization, showing substantially reduced $P_{r}$ in thinner ${\mathrm{BaTiO}}_{3}$ films. (c) Transient depolarization fields extracted from the relaxation dynamics, all converging to the same equilibrium value below the coercive field. Error bars represent uncertainty propagated from repeated measurements per sample; bars are largest at long delay times where the polarization relaxation rate approaches zero. (d) Hysteresis behavior from phase‐field simulations with the inclusion of the equilibrium depolarization obtained experimentally. The static depolarization induces reverse‐domain formation, but does not capture the experimental slanting and loop shape.

As can be done in traditional forward‐switching studies, the polarization transients can be analyzed within a nucleation‐and‐growth framework that relates the rate of polarization change to the driving electric field [31]:

(1) $$\begin{array}{cc} \frac{dP(t)}{dt} & =2\pi nu\left(t\right)P_{sw}\int_{0}^{t}u\left(t^{\prime }\right)dt^{\prime } \end{array}$$

(2) $$\begin{array}{cc} u(t) & =u_{\circ }\exp\left[-E_{a}/E_{FE}\left(t\right)\right], \end{array}$$

where P is the polarization, $n\approx 10^{14}\;cm^{-2}$ is the areal density of nucleation sites (based on previous modeling [31]), u is the domain‐wall velocity, $u_{\circ }\approx 3.82\times 10^{4}\;cm\;s^{-1}$ is the infinite‐field domain‐wall velocity [29], $P_{sw}$ is the switchable polarization, $E_{a}\approx 12E_{c}$ is the activation field associated with domain‐wall motion (in the range common to all ferroelectrics [18] and in agreement with previous study of ${\mathrm{BaTiO}}_{3}$ [29, 34]), and $E_{FE}$ is the electric field across the ferroelectric. Since polarization reversal occurs with no applied field, $E_{FE}$ is simply the depolarization field ($E_{d}$).

Numerically solving this system (Supporting Information) yields the transient depolarization field driving reverse‐domain formation (Figure 2c). A striking result emerges: despite variations in film thickness and final remanent polarization, the transient depolarization fields converge to a common equilibrium value across all heterostructures. Initially, the depolarization field exceeds the coercive field before decreasing continuously as relaxation proceeds. This evolution is consistent with the observed power‐law decay of polarization, reflecting a diminishing driving force as domain‐wall motion approaches equilibrium. This convergence suggests that the equilibrium depolarization field is governed by the intrinsic switching energetics of ${\mathrm{BaTiO}}_{3}$ – independent of film thickness. What varies as the system is scaled down, however, is the remanent polarization at which this equilibrium is reached, reflecting a shift in the balance between the ferroelectric layer and its boundary conditions.

To test whether the equilibrium field alone accounts for the thickness‐dependent polarization loss, phase‐field simulations (Experimental Section) were carried out for a 50‐nm‐thick ${\mathrm{BaTiO}}_{3}$ film, a representative thickness that retains measurable remanence while exhibiting strong depolarization effects in experiments. The experimentally extracted equilibrium depolarization field was implemented as a static, spatially uniform internal field opposing the polarization (Figure 2d). While this approach successfully reaches the correct final state (qualitatively reproducing a reduction in remanent polarization and reverse‐domain formation at zero applied field) the mechanism by which this is achieved is at odds with experiment. The introduction of a static depolarization field in the simulation results in narrowed loops with abrupt switching at a reduced coercive field, in contrast to experimental measurements which demonstrate a loss of remanent polarization, consistent coercive field, and slanted loops, with switching events becoming broadened. The time‐resolved dynamics reveal the flaw: the depolarization field is not static but evolves with the polarization state. Thus, a more accurate model of the electrostatic origins of the depolarization field is required to properly describe not only the reduced polarization stability but the path the system takes to reach that equilibrium.

### 2.3 Electrostatic Origin of Depolarization Fields

To identify the physical origin of the depolarization fields, a simple electrostatic model is introduced. The ferroelectric heterostructures are modeled as a series combination of capacitive elements representing the ferroelectric film and the electrode screening interfaces (Figure 3a). Assigning capacitance to each component allows derivation of a constitutive equation relating the depolarization field to the interfacial capacitance and the ferroelectric thickness:

(3) $$E_{d}=2P\frac{A}{C_{scn}t_{FE}}$$

where $E_{d}$ is the depolarization field, P is the average ferroelectric polarization, $C_{scn}$ is the capacitance of the electrode screening region, A is the device area, and $t_{FE}$ is the ferroelectric thickness. Because the ${\mathrm{BaTiO}}_{3}$/${\mathrm{SrRuO}}_{3}$ interfaces are nominally identical across samples, $C_{scn}$ should be universal.

**FIGURE 3:**
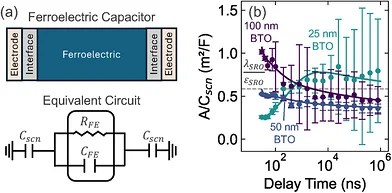
Electrostatic model of depolarization and extraction of interfacial screening capacitance. (a) Schematic illustration of the ferroelectric capacitor heterostructure, highlighting the interfacial screening regions and their representation in an equivalent series‐capacitance circuit. (b) Extracted inverse‐capacitance density of the interfacial screening region for each ${\mathrm{BaTiO}}_{3}$ thickness, showing similar values across all devices and agreement with expectations for ${\mathrm{SrRuO}}_{3}$ screening.

Using the experimentally extracted polarization and depolarization field $\left(P,E_{d}\right)$ pairs (Figure 2b,c), Equation (3) yields an effective‐screening capacitance for each device (Figure 3b). All samples collapse to similar values of $C_{scn}$ within uncertainty, confirming that the differences in depolarization behavior arise predominantly from the ferroelectric thickness and not from variations in interface quality. The inferred $C_{scn}$ agrees (within error) with expectations from ${\mathrm{SrRuO}}_{3}$ screening based on a Thomas‐Fermi screening length of $\lambda_{s}\approx 0.5$ Å and a dielectric constant of $\varepsilon_{e}\approx 10\varepsilon_{0}$ [35, 36].

This observation allows the interfacial screening to be recast as an effective capacitance per unit area,

(4) $$\frac{C_{scn}}{A}\equiv \frac{\varepsilon_{e}}{\lambda_{s}},$$

allowing the classical screening‐based depolarization expression [13]

(5) $$E_{d}=\frac{P}{\varepsilon_{FE}}\left(\frac{2\varepsilon_{FE}/t_{FE}}{\varepsilon_{e}/\lambda_{s}+2\varepsilon_{FE}/t_{FE}}\right)$$

to be rewritten in a more transparent circuit form:

(6) $$E_{d}=\frac{P}{\varepsilon_{FE}}\left(\frac{C_{FE}}{C_{scn}/2+C_{FE}}\right),$$

where $C_{FE}$ is the ferroelectric‐layer capacitance. This circuit representation reveals the system‐level nature of depolarization wherein the field depends on the ratio of the ferroelectric capacitance to the total series capacitance, suggesting that depolarization can be systematically engineered through modification of the electrostatic boundary conditions. In particular, the introduction of additional series capacitances at the ferroelectric‐electrode interface provides a direct means to control the depolarization field and, consequently, the stability of the remanent polarization.

### 2.4 Tuning Depolarization via Interfacial Capacitance

The screening model establishes that depolarization fields depend on the ratio of ferroelectric to total series capacitance. An immediate implication is that depolarization can be deliberately tuned by modifying the series capacitance at the interfaces. If interfacial capacitance can be engineered, so can the stability of the remanent polarization. To test this premise experimentally, thin dielectric buffer layers were introduced at the ${\mathrm{BaTiO}}_{3}$/${\mathrm{SrRuO}}_{3}$ interfaces to systematically adjust the interfacial capacitance in a controlled and well‐quantified manner.

To modulate the interfacial electrostatics, heterostructures consisting of 30 nm ${\mathrm{SrRuO}}_{3}$/1.2 or 3.6 nm ${\mathrm{SrTiO}}_{3}$/50 nm ${\mathrm{BaTiO}}_{3}$/1.2 or 3.6 nm ${\mathrm{SrTiO}}_{3}$/30 nm ${\mathrm{SrRuO}}_{3}$ were grown (Experimental Section), wherein the incorporation of ultrathin ${\mathrm{SrTiO}}_{3}$ layers is achieved at both ferroelectric‐electrode interfaces. X‐ray diffraction ω–2θ line scans and reciprocal space maps confirm coherent strain is preserved in all buffer‐layer configurations (Figure S2). ${\mathrm{SrTiO}}_{3}$ is an ideal choice for such an experiment because its large room‐temperature permittivity ($\approx 300\varepsilon_{0}$) [37] allows very thin layers to introduce sufficiently large capacitances as to not dominate the overall circuit, while its structural compatibility with ${\mathrm{BaTiO}}_{3}$ avoids extrinsic degradation mechanisms. The 1.2 and 3.6 nm layers provide inverse‐capacitance densities of 0.44 and 1.32 $m^{2}$ $F^{-1}$, respectively – comparable to the effective screening capacitance previously extracted for the ${\mathrm{SrRuO}}_{3}$ electrodes. Thus, the dielectric layers are expected to significantly influence the polarization stability through the same physical mechanism as reducing the ferroelectric thickness: both modify the ferroelectric‐to‐interfacial capacitance ratio ($C_{FE}/C_{scn}$) and thereby amplify depolarization fields.

Experimentally, increasing the ${\mathrm{SrTiO}}_{3}$ thickness produces a systematic reduction in the remanent polarization (Figure S5a) and a marked increase in the hysteresis‐loop slant (Figure 4a), closely mimicking the behavior observed during ${\mathrm{BaTiO}}_{3}$ thickness scaling. This similarity suggests a common underlying mechanism rooted in electrostatics rather than intrinsic changes to the ferroelectric. PUND measurements reinforce this interpretation: the remanent polarization decreases systematically with increasing ${\mathrm{SrTiO}}_{3}$ thickness, while the depolarization field extracted from the relaxation dynamics converges to the same equilibrium value across all buffer‐layer thicknesses (Figure 4b). Applying the simple electrostatic model for depolarization to these data yields a corresponding decrease in the effective interfacial capacitance, matching the expectation from a simple series‐capacitance model:

(7) $$\frac{1}{C_{ser}}=\frac{2}{C_{scn}}+\frac{2}{C_{STO}},$$

where $C_{ser}$ is the total interfacial capacitance, $C_{scn}$ is the ${\mathrm{SrRuO}}_{3}$ screening capacitance, and $C_{STO}$ is the capacitance of the ${\mathrm{SrTiO}}_{3}$ buffer layer (Figure S5b). The experimental trend follows this series rule closely, providing quantitative confirmation that the dielectric layers influence polarization stability by modifying the interfacial electrostatics while leaving the equilibrium depolarization field supported by ${\mathrm{BaTiO}}_{3}$ unchanged.

**FIGURE 4:**
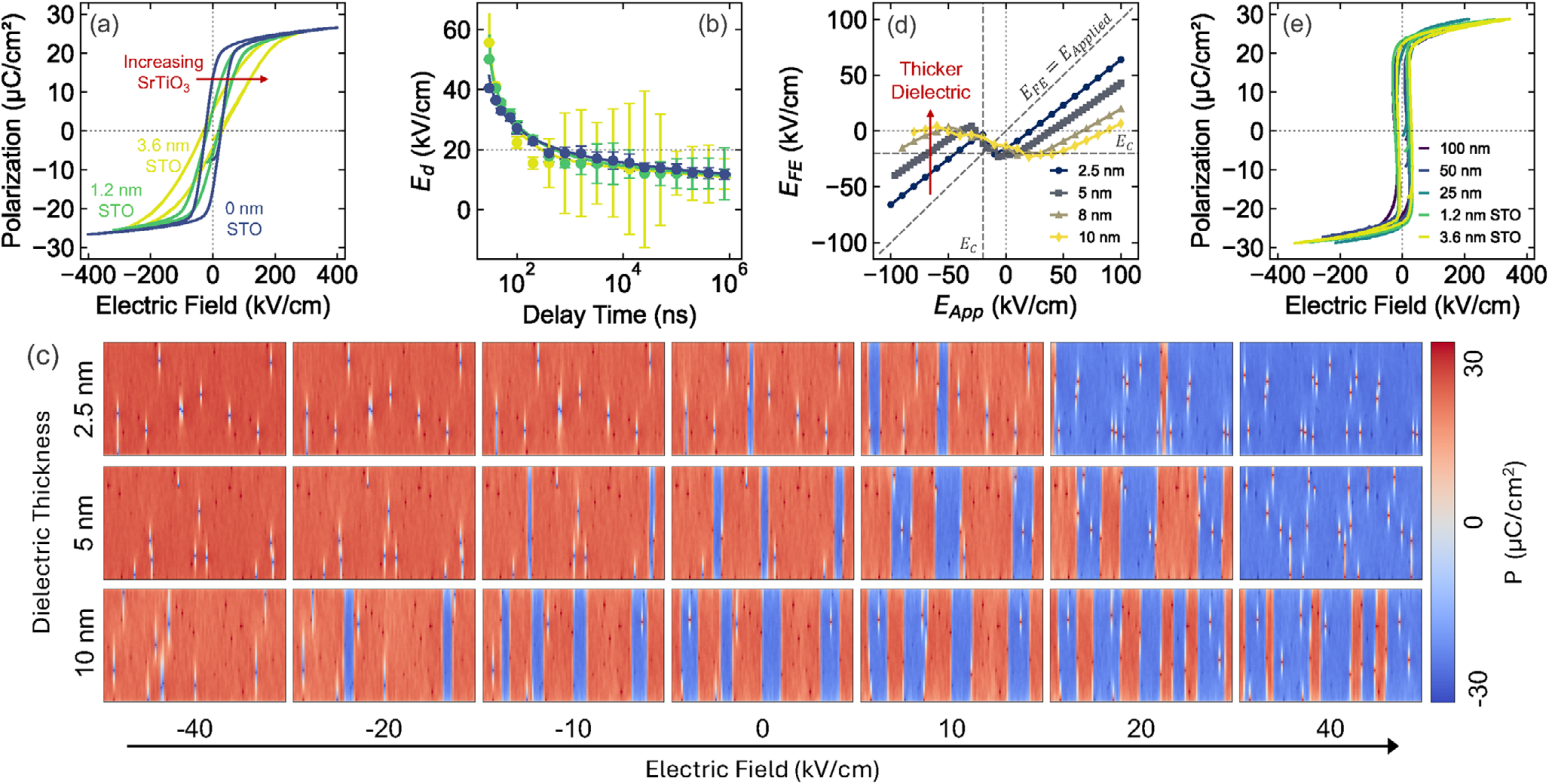
Tuning depolarization through interfacial dielectric buffer layers. (a) Polarization‐electric field hysteresis loops for 50‐nm‐thick ${\mathrm{BaTiO}}_{3}$ devices with 0, 1.2, and 3.6 nm ${\mathrm{SrTiO}}_{3}$ buffer layers, showing reduced $P_{r}$ and increased loop slant as the dielectric layer thickness increases. (b) Extracted depolarization field transients, again showing convergence to a single equilibrium value despite variation of electrostatic boundary conditions. (c) Domain maps as a function of applied electric field from phase‐field simulations for 50‐nm‐thick ${\mathrm{BaTiO}}_{3}$ films with dielectric layers of increasing thickness at the interfaces. From top to bottom, dielectric layers of 2.5, 5, and 10 nm. (d) Increasing divergence between the applied field ($E_{App}$) and the field across the ferroelectric ($E_{FE}$) with increasing dielectric thickness in the phase‐field simulations. (e) Experimental polarization‐electric field hysteresis loops corrected for the field across the ferroelectric layer, collapsing all devices onto the same intrinsic ${\mathrm{BaTiO}}_{3}$ response.

Phase‐field simulations provide a microscopic view of this behavior. Each simulated stack consisted of a 50‐nm‐thick ${\mathrm{BaTiO}}_{3}$ layer sandwiched by symmetric dielectric layers of thickness 2.5, 5, 8, or 10 nm. The dielectric layers were treated as linear high‐permittivity media ($\varepsilon_{r}=\varepsilon_{\text{STO}}$). In this configuration, the only systematic variation across the simulation series is the extrinsic series capacitance, while the intrinsic ferroelectric free‐energy landscape remains fixed. The simulated domain maps reveal a clear broadening of the switching transition with increasing dielectric thickness (Figure 4c). For thin dielectric buffers, polarization reversal occurs over a narrow field range, with reverse domains nucleating and propagating rapidly. As the dielectric‐layer thickness increases, switching initiates at lower fields but proceeds more gradually: reverse domains nucleate earlier, grow more slowly, and coalesce over a substantially wider field window. This broadening mirrors the experimentally observed increase in loop slant and reduction in remanent polarization, reflecting the enhanced influence of depolarization as the interfacial capacitance decreases.

Analysis of the Landau, gradient, and electrostatic energies in the phase‐field simulations reveals that the intrinsic switching landscape of ${\mathrm{BaTiO}}_{3}$ remains essentially unchanged across all dielectric‐buffered configurations (Figure S6). The dominant differences arise instead from how the electric field is redistributed within the heterostructures (Figure 4d). As the dielectric layers are made thicker, a larger fraction of the applied voltage drops across the series layers, and a correspondingly smaller fraction is delivered to the ferroelectric. Simultaneously, the depolarization field opposing the polarization increases for a given polarization state.

This voltage redistribution can be expressed through the effective field acting on the ferroelectric of the form,

(8) $$E_{FE}=\frac{V_{app}}{t_{FE}}\left(\frac{C_{ser}}{C_{ser}+C_{FE}}\right)-\frac{P}{\varepsilon_{FE}}\left(\frac{C_{FE}}{C_{ser}+C_{FE}}\right),$$

which captures two distinct contributions to the deviation between the applied field and the field experienced by the ferroelectric layer. The first term represents the voltage drop across the interfacial series capacitances, resulting in an overall weaker field response of the ferroelectric layer. The second term captures the development of the depolarization field, which opposes the polarization state and further increases the divergence between the applied field and the depolarization field. Importantly, because the depolarization field depends on the polarization state, this opposing field evolves dynamically during switching, producing distinct effects on the forward and reverse switching processes.

When switching from a poled state, the depolarization field initially assists in overcoming the switching barrier, driving the field experienced by the ferroelectric layer toward the coercive field and enabling the nucleation of reverse‐domains. As the applied field is swept further, domain growth evolves the depolarization field in a way that clamps the field experienced by the ferroelectric layer near the coercive field – a feedback between domain growth and depolarization – sustaining switching over a wider applied field range until switching is nearly complete. This produces broad plateaus in the simulated electric field‐polarization trajectories, the width of which grows with dielectric thickness as depolarization fields become more influential. After net polarization reversal, the depolarization field opposes further switching, effectively “stretching‐out” the switching process more strongly with lower interfacial capacitances.

When experimental polarization‐electric field hysteresis loops are replotted using the field experienced by the ferroelectric layer ($E_{FE}$) from Equation (8), using permittivity values from dielectric characterization of capacitor devices (Figures S8, S9), all devices collapse onto a single curve regardless of the ${\mathrm{BaTiO}}_{3}$ or dielectric‐buffer layer thickness (Figure 4e). This universal collapse establishes that dielectric buffering does not modify the intrinsic switching physics of ${\mathrm{BaTiO}}_{3}$, but instead redistributes the applied voltage between the external drive and the internally generated depolarization fields. Once this voltage partitioning is accounted for, the ferroelectric response is universal across all architectures.

### 2.5 Defects and the Switching Pathway

Within this domain‐mediated switching framework, the nature and density of defects play a secondary but consequential role in shaping polarization dynamics. While defects (generally) do not alter the depolarization field itself, they strongly influence the pathways through which depolarization‐driven relaxation proceeds. As ferroelectric materials are optimized toward lower coercive fields and switching voltages, the absence of barriers to reverse‐domain formation and propagation renders polarization increasingly susceptible to depolarization‐driven relaxation. In this regime, the controlled introduction of selected defects offers a pathway to restore stability without substantially compromising low‐voltage operation. This perspective parallels silicon CMOS fabrication, in which extreme material purification enables the deliberate reintroduction of specific dopants and defects to control device functionality. In other words, to achieve ultra‐low coercive fields it may be necessary to produce low‐defect films, only to be required to reintroduce desired defects to stabilize polarization against depolarization fields and backswitching.

Phase‐field simulations provide a means to explore this balance by independently tuning the density and character of defects in ways that are difficult to isolate experimentally. Two representative classes of defects are considered here: heterogeneous nucleation centers, which lower the barrier for polarization reversal, and pinning sites, which impede lateral domain‐wall motion. Systematic variation of these defect populations reveals that they influence polarization stability through distinct and separable mechanisms. Increasing the nucleation‐site density does not substantially alter the coercive field (Figure 5a). A higher density of nucleation sites does, however, increase the volume of the ferroelectric that nucleates reverse domains at the onset of relaxation. This larger initial volume participating in reversal under the depolarization field leads to a greater initial drop in polarization with increasing nucleation site density. The coercive field remains insensitive to nucleation‐site density because the barrier to domain‐wall propagation is unchanged. In contrast, pinning sites primarily influence the growth stage of switching. By impeding lateral domain‐wall motion, pinning sites increase the effective barrier for domain growth, resulting in a modest increase in coercive field with pinning‐site density (Figure 5b). This suppresses depolarization‐driven reverse‐domain formation by inhibiting domain‐wall motion under internal fields, stabilizing polarized regions and supporting higher remanent polarization.

**FIGURE 5:**
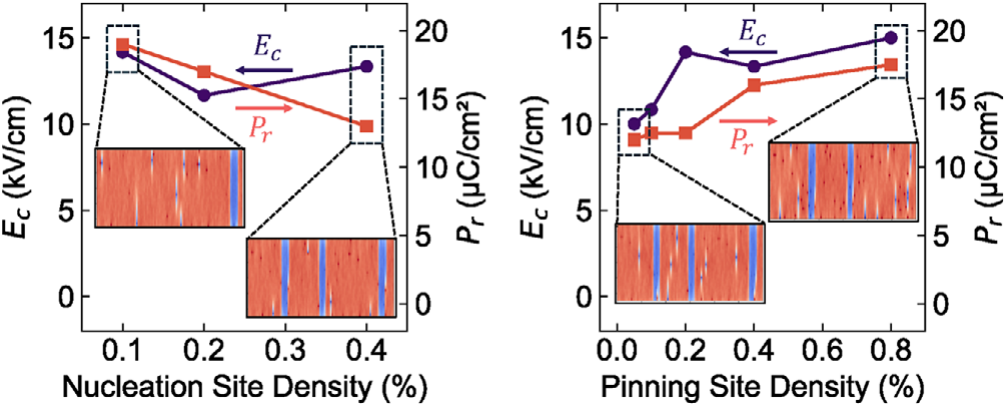
Defect populations independently control coercive field and remanent polarization. Extracted coercive field ($E_{C}$) and remanent polarization ($P_{r}$) from phase‐field simulations sweeping nucleation‐ and pinning‐site densities. (a) Increasing nucleation‐site density leads to a decrease in remanent polarization with minimal impact on coercive field. (b) Higher pinning‐site densities correspond to increases in both coercive field and remanent polarization due to local inhibition of domain‐wall motion.

Once it is realized that depolarization fields are (ultimately) governed by electrostatic boundary conditions, further improvements in polarization stability must arise from controlling the relaxation pathways rather than modifying the depolarization field itself. The simulations demonstrate that tuning defect populations provides such control, with pinning sites enhancing remanent polarization. Ion implantation or bombardment offers a practical means of tuning defect densities in perovskite ferroelectrics [38, 39]. At low defect densities, introduced defects primarily act as domain‐wall pinning sites, leading to modest increases in coercive field and enhanced remanent polarization stability. This approach thus provides a practical route to improve polarization stability in ultrathin ferroelectric devices through controlled modification of switching energetics.

## 3 Conclusions

In ultrathin ${\mathrm{BaTiO}}_{3}$ heterostructures, the reduction of remanent polarization arises from depolarization‐driven domain formation imposed by electrostatic boundary conditions, rather than from suppression of the polar distortion. Fast‐relaxation measurements reveal depolarization fields that evolve dynamically and converge to a thickness‐independent equilibrium value. A simple electrostatic model establishes that these fields are set by the effective series capacitance associated with incomplete electrode screening, a picture which quantitatively accounts for thickness scaling and is validated by dielectric‐buffer experiments. Phase‐field simulations confirm that modifying interfacial capacitance reshapes switching by redistributing internal fields without altering the intrinsic ferroelectric energy landscape.

These results establish depolarization as a system‐level effect governed by boundary conditions of the heterostructure, rather than an intrinsic limitation of the ferroelectric material itself. This reframing carries practical consequences: polarization stability in ultrathin ferroelectrics can be engineered through two complementary handles–interfacial design to modify the electrostatic driving force for instability, and defect management to govern the relaxation pathways through which that instability unfolds. Advancing ultrathin ferroelectric devices toward low‐voltage, non‐volatile operation will likely require simultaneous optimization on both fronts.

## 4 Experimental Section

### 4.1 Growth of Thin‐Film Heterostructures

All films produced in this work were grown by pulsed‐laser deposition in a dedicated, custom‐designed deposition chamber. All films were grown using an excimer laser (LPX 300, KrF excimer laser with λ=248 nm; Coherent, Inc.). For all depositions, the target‐to‐substrate distance was controlled to be 5.5 cm. All temperatures reported refer to those measured by a thermocouple inserted in the substrate‐heater block (and, as such, may not represent the exact substrate temperature). Substrates were affixed to the resistive heater with silver paint (PELCO Conductive Silver Paint, No. 16062; Ted Pella, Inc.) and subsequently heated to 100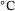 and held for five minutes to drive‐off the solvent and cure the silver paint, assuring robust thermal contact. Prior to deposition, the chamber was evacuated to a base pressure of at least $5\times 10^{-6}$ Torr.

For this work, the focus was on thin‐film heterostructures of the form 30 nm ${\mathrm{SrRuO}}_{3}$/25–100 nm ${\mathrm{BaTiO}}_{3}$/30 nm ${\mathrm{SrRuO}}_{3}$ which were grown on ${\mathrm{GdScO}}_{3}$ (110) single‐crystal substrates (CrysTec, GmbH). All film thickness values were established by X‐ray diffraction. All layers were grown in situ without breaking the vacuum. For the deposition, substrates were first ramped to the growth temperature for the bottom electrode (680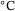 at 10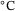 $\min^{-1}$) in a dynamic oxygen pressure of 100 mTorr. During the ramp‐up phase, the targets were pre‐ablated to clean the surfaces. The pre‐ablation process consisted of, for the ${\mathrm{SrRuO}}_{3}$ target, 1000 shots at a laser fluence of 1.1 J ${\mathrm{cm}}^{-2}$, and for the ${\mathrm{BaTiO}}_{3}$ target, 1000 shots at a laser fluence of 1.5 J ${\mathrm{cm}}^{-2}$. Upon reaching the growth temperature, the system was allowed to reach thermal equilibrium over 5 min prior to the start of the deposition process.

The bottom ${\mathrm{SrRuO}}_{3}$ layer was deposited from a ceramic target of the same composition (Praxair Surface Technologies), the material was deposited at a laser fluence of 1.1 J ${\mathrm{cm}}^{-2}$ and a laser repetition rate of 10 Hz in a dynamic oxygen pressure of 100 mTorr. Following completion of the bottom electrode growth, the substrate was then cooled to 630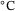 at 10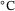 $\min^{-1}$ for the growth of the next layer. Upon reaching the new temperature, the system was again allowed to equilibrate for 5 min and just prior to the resumption of growth, the dynamic oxygen pressure was reduced to 60 mTorr, and the 25–100 nm ${\mathrm{BaTiO}}_{3}$ ferroelectric layer was deposited from a ceramic target of the same composition (Praxair Surface Technologies) at a laser fluence of 1.5 J ${\mathrm{cm}}^{-2}$ and a laser repetition rate of 2 Hz. Following the growth of this layer, the substrate was heated back to 680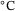 at 10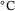 $\min^{-1}$ in a dynamic oxygen pressure of 100 mTorr. Upon reaching the new temperature, the system was again allowed to equilibrate for 5 min and the 30 nm ${\mathrm{SrRuO}}_{3}$ top electrode was deposited from the same target and under the same laser conditions as the bottom layer. Following completion of the deposition, the chamber was flooded to a static oxygen pressure of ≈700 Torr and the heterostructure was cooled to room temperature at a rate of 5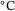 $\min^{-1}$.

For heterostructures incorporating ${\mathrm{SrTiO}}_{3}$ buffer layers at the ferroelectric‐electrode interfaces, the growth sequence was modified as follows: after reaching the deposition temperature of 630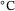 and allowing for thermal equilibration, a thin ${\mathrm{SrTiO}}_{3}$ layer (1.2 or 3.6 nm) was deposited on the bottom ${\mathrm{SrRuO}}_{3}$ electrode from a single‐crystal target at a laser fluence of 1.4 J ${\mathrm{cm}}^{-2}$ and a repetition rate of 2 Hz in 60 mTorr oxygen pressure. Immediately following deposition of this buffer layer, the ${\mathrm{BaTiO}}_{3}$ ferroelectric layer was deposited under the standard conditions (1.5 J ${\mathrm{cm}}^{-2}$, 2 Hz). Upon completion of the ${\mathrm{BaTiO}}_{3}$ deposition, a second ${\mathrm{SrTiO}}_{3}$ buffer layer of identical thickness was deposited at the same conditions (1.4 J ${\mathrm{cm}}^{-2}$, 2 Hz). The substrate was then heated to 680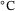 at 10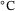 $\min^{-1}$, the system was equilibrated for 5 minutes, and the top ${\mathrm{SrRuO}}_{3}$ electrode was deposited under the standard conditions. The samples were subsequently cooled and processed as described above.

### 4.2 X‐Ray Diffraction

X‐ray diffraction measurements including ω–2θ line scans were used to characterize the phase, structure, epitaxy, and strain state of the films. Measurements were completed on a Malvern Panalytical X'${\mathrm{Pert}}^{3}$ equipped with a 1.8 kW sealed source. All laboratory‐based diffraction studies were completed using copper $K_{\alpha }$ radiation (λ=1.5406 Å). The incident beam optics included a hybrid two‐bounce Ge (220) monochromator and a 1/2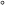 divergence slit. After interacting with the sample, the diffracted X‐rays were collected with a pixelated area detector (PIXcel3D) operating in 0D (point detection) mode for line scans. Receiving optics were configured to provide a 0.275 mm active channel length at the detector. Symmetric ω–2θ line scans were performed with a step size of 0.01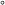 and a dwell time of 0.3–0.6 seconds per step. For asymmetric measurements including reciprocal space mapping studies of asymmetric reflections (e.g., in this work that includes the 103‐ and 332‐diffraction conditions), the data were collected by scanning the ω axis while collecting a range of 2θ angles on the pixelated detector. Prior to all scans, the ω, Z, and χ axes were aligned to a strong substrate diffraction condition (typically the ${\mathrm{GdScO}}_{3}$ 220‐diffraction condition).

### 4.3 Dielectric and Ferroelectric Measurements

All dielectric and ferroelectric measurements were performed at room temperature. Polarization‐electric field hysteresis loops were measured using a Radiant Technologies Precision Multiferroic II tester employing virtual‐ground charge integration. Measurements used bipolar triangular waveforms at 1 kHz to balance leakage suppression with RC‐limited bandwidth. Each hysteresis loop was acquired using a double‐bipolar waveform following a preset poling pulse with a delay of 1000 ms. For ${\mathrm{BaTiO}}_{3}$ capacitors, field amplitudes of 100–200 kV ${\mathrm{cm}}^{-1}$ typically ensured polarization saturation without getting close to dielectric breakdown. Remanent polarization, saturation polarization, and coercive field were determined by integration of the displacement current.

Pulsed‐switching measurements using the PUND technique were employed to separate ferroelectric‐switching current from non‐switching contributions such as leakage and displacement current. The PUND sequence consists of four voltage pulses (i.e., P, U, N, and D) of identical amplitude and duration. Switching currents are isolated by subtracting the non‐switching response (U from P, and D from N), and the switched polarization is obtained by time integration of the resulting transient. In the case of films exhibiting unstable remanent polarization, as studied here, the difference between pulses changes with delay time, giving access to the dynamics of the polarization‐reversal process. Nanosecond‐scale switching dynamics were captured using a Berkeley Nucleonics 865 pulse generator (70 ps rise time) and a Tektronix TDS6604 oscilloscope (6 GHz bandwidth), enabling faithful acquisition of sub‐nanosecond switching transients and polarization retention measurements over timescales from 30 ns to 800 μs. PUND pulse amplitudes of 300 kV ${\mathrm{cm}}^{-1}$ were applied across all film thicknesses, ensuring polarization saturation prior to each relaxation measurement.

Dielectric permittivity ($\varepsilon^{\prime }$) and loss tangent (tanδ) were measured on a Keysight E4980A LCR meter connected to a probe station. Data were collected under a 10 kHz AC oscillation with an amplitude of 100 mV while sweeping DC bias between −200 kV ${\mathrm{cm}}^{-1}$ and 200 kV ${\mathrm{cm}}^{-1}$ in both the upward and downward direction.

### 4.4 XAS and XLD Measurements

Measurements were conducted at beamline 4.0.2 of the Advanced Light Source, Lawrence Berkeley National Laboratory. Spectra were acquired in total electron yield (TEY) mode under ultrahigh vacuum with a probing depth of approximately 3–5 nm [40, 41], providing sensitivity to the near‐surface region of the films. Grazing‐incidence geometry was employed ($\theta =10^{\circ }$) with linear polarization control over the incident X‐rays to enable distinction between in‐plane and out‐of‐plane absorption.

Spectra were collected at the titanium $L_{2,3}$ edges (450–494 eV) and the oxygen K edge (510–594 eV), spanning an energy range from pre‐edge through post‐edge. In one polarization configuration the incident electric‐field vector lies entirely within the film plane and yields the in‐plane response $I_{ab}$ directly; in the other it lies 10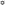 from the polar c axis and measures a mixture of the two responses,

(9) $$I_{meas}=cos^{2}\theta \cdot I_{c}+sin^{2}\theta \cdot I_{ab}.$$

Because $I_{ab}$ is obtained without admixture, the out‐of‐plane response is recovered as

(10) $$I_{c}=\left(I_{meas}-sin^{2}\theta \cdot I_{ab}\right)/cos^{2}\theta$$

and the linear dichroism response is constructed as

(11) $$\Delta I\left(E\right)=I_{c}\left(E\right)-I_{ab}\left(E\right),$$

which reflects the anisotropic absorption arising from the ferroelectric structural distortion.

Data processing consisted of pre‐edge background subtraction using a linear fit to the pre‐edge region and post‐edge normalization to the edge jump at high energy, yielding normalized absorption spectra μ(E) for each polarization, from which ΔI(E) was constructed. Measurements were performed on uncapped ${\mathrm{BaTiO}}_{3}$ samples with nominal thicknesses ranging from 3 nm to 100 nm, and on companion heterostructures capped with 3 nm of ${\mathrm{SrRuO}}_{3}$, which are compared with the uncapped series in the Supporting Information. Full details of the measurement geometry, data reduction, and the treatment of the ${\mathrm{SrRuO}}_{3}$ contribution to the capped heterostructures are additionally given in the Supporting Information.

### 4.5 Phase‐Field Simulations

Phase‐field simulations were performed using the code package *FerroX* [42], which is a time‐dependent Ginzburg‐Landau model for ${\mathrm{BaTiO}}_{3}$ incorporating Landau free‐energy, gradient‐energy, and electrostatic‐energy terms [43]. The total free‐energy density is

(12) $$F=f_{\mathrm{Landau}}+f_{\mathrm{grad}}+f_{\mathrm{elec}},$$

where the Landau free‐energy density is

(13) $$f_{\mathrm{Landau}}=\frac{1}{2}\alpha_{1}P_{z}^{2}+\frac{1}{4}\alpha_{11}P_{z}^{4}+\frac{1}{6}\alpha_{111}P_{z}^{6},$$

with $\alpha_{1}$, $\alpha_{11}$, and $\alpha_{111}$ being the standard Landau coefficients for ${\mathrm{BaTiO}}_{3}$. The gradient‐energy density is

(14) $$f_{\mathrm{grad}}=\frac{1}{2}G_{11}{\left(\frac{\partial P_{z}}{\partial z}\right)}^{2}+\frac{1}{2}G_{44}\left[{\left(\frac{\partial P_{z}}{\partial x}\right)}^{2}+{\left(\frac{\partial P_{z}}{\partial y}\right)}^{2}\right],$$

where $G_{11}$ governs thickness variation and $G_{44}$ controls lateral domain‐wall energy. The electrostatic energy density is

(15) $$f_{\mathrm{elec}}=E_{z}P_{z},$$

with the electric field obtained self‐consistently from Gauss's law,

(16) $$\nabla \cdot \left(\varepsilon_{0}\nabla \phi +P\right)=0.$$

In‐plane polarization components are neglected in these simulations as, in the real system, compressive biaxial epitaxial strain restricts the polar axis to the out‐of‐plane c‐direction, while the reduction in computational overhead permits simulation domains approaching 1 μm in lateral extent, enabling more accurate capture of the mesoscale impact of domain formation on polarization – as would be measured in a device. The boundary conditions at the ferroelectric interfaces are implemented using a finite extrapolation length λ,

(17) $${\left.\frac{\partial P_{z}}{\partial z}\right|}_{\mathrm{interface}}\propto \frac{P_{z}}{\lambda },$$

which enforces continuity of the normal component of the displacement field across the interface and allows interfacial potentials to develop self‐consistently. Model parameters are listed in Table 1.

**TABLE 1:** Phase‐field model parameters used in simulations of ${\mathrm{BaTiO}}_{3}$ thin films.

**Parameter**	**Value**	**Description**
*Landau Coefficients*		
$\alpha_{1}$ ($10^{7}$ $m^{2}N$/$C^{2}$)	−1.8558	Second‐order
$\alpha_{11}$ ($10^{8}$ $m^{6}N$/$C^{4}$)	−2.097	Fourth‐order
$\alpha_{111}$ ($10^{10}$ $m^{10}N$/$C^{6}$)	1.0352	Sixth‐order
*Gradient Energy Coefficients*		
$G_{11}$ ($10^{-10}$ $m^{3}V$/C)	2	Out‐of‐plane
$G_{44}$ ($10^{-11}$ $m^{3}V$/C)	1	In‐plane
*Boundary Conditions*		
$\varepsilon_{\mathrm{FE}}$	600$\varepsilon_{0}$	Ferroelectric permittivity
$\varepsilon_{\mathrm{DE}}$	300$\varepsilon_{0}$	Dielectric permittivity
λ	5 nm	Extrapolation length

Nucleation and pinning defects are introduced as spatially localized perturbations to the Landau coefficients. Defect locations are determined using a pseudo‐random number generator with a fixed random seed to ensure consistency across trials. Nucleation sites fix polarization to saturation values; pinning sites increase the $\alpha_{1}$ coefficient magnitude tenfold, raising the local nucleation barrier. These approaches enable controlled variation of defect density and type to examine their influence on switching dynamics and polarization stability. Calibration of this defect‐mediated switching framework, including comparison between homogeneous and nucleation‐driven reversal pathways, is provided in the Figure S7.

The phase‐field computation is coded as a structured–grid, finite–difference, time–domain multiphysics solver built on the *AMReX* framework, which decomposes the 3D domain into non–overlapping grid patches mapped to MPI ranks and runs portably on CPUs or GPUs via an MPI+X approach (e.g., CUDA), minimizing host–device transfers and relying mainly on ghost–cell exchanges between ranks [42]. The simulation advances in time by evolving the polarization field with a second–order temporal integrator (a predictor step with an optional corrector), and after each polarization update it recomputes electrostatic field self–consistently. *FerroX* uses a fixed–point loop that “lags” the potential dependence and iterates the Poisson solve to convergence (stopping when the mean cellwise change drops below a specified tolerance, e.g., $10^{-5}$, typically in a few iterations). Electrostatics is solved with *AMReX*'s geometric multigrid (MLMG) solver, using periodic boundary conditions in–plane and Dirichlet conditions through the stack thickness to impose applied voltages, which makes the overall workflow a tightly coupled, self–consistent time–stepping loop suitable for device–scale simulations.

## Author Contributions

T.L.: Conceptualization, formal analysis, investigation, methodology, visualization, writing – original draft, writing – review and editing. P.Ku.: Investigation, methodology, software. C.K.: Investigation. P.Ka.: Investigation. P.S.: Supervision, writing – review and editing. Z.Y.: Methodology, software, supervision, writing – review and editing. L.W.M.: Conceptualization, funding acquisition, resources, supervision, writing – original draft, writing – review and editing.

## Conflicts of Interest

The authors declare no conflicts of interest.

## Supporting information


**Supporting File**: advs78103‐sup‐0001‐SuppMat.pdf.

## Data Availability

All data needed to evaluate the conclusions in this paper are available from the authors upon reasonable request.
