# Supplementary material for: Unraveling the Effects of Depolarization in Ultrathin Barium Titanate
Source: Adv Sci (Weinh). 2026 Oct 31:e78103. Online ahead of print. doi: 10.1002/advs.78103 (PMC13723724; doi:10.1002/advs.78103)
Supplement: Supplementary file 1 [file ADVS-9999-e78103-s001.pdf]

*Supporting Information for*

# Unraveling the Effects of Depolarization in Ultrathin Barium Titanate

*Thomas Lee,*[1,2,3] *Prabhat Kumar,*[2] *Christoph Klewe,*[4] *Pravin Kavle,*[1,2] *Padraic Shafer,*[5] *Zhi (Jackie) Yao,*[6] *Lane W. Martin*[2,3,7*]

[1] Department of Materials Science and Engineering, University of California, Berkeley, California 94720, USA
[2] Materials Sciences Division, Lawrence Berkeley National Laboratory, Berkeley, California 94720, USA
[3] Rice Advanced Materials Institute, Rice University, Houston, Texas 77005, USA
[4] Advanced Light Source, Lawrence Berkeley National Laboratory, Berkeley, California 94720, USA
[5] National Synchrotron Light Source II, Brookhaven National Laboratory, Upton, New York 11973, USA
[6] Applied Mathematics and Computational Research Division, Lawrence Berkeley National Laboratory, Berkeley, California 94720, USA
[7] Departments of Materials Science and NanoEngineering, Chemistry, and Physics and Astronomy, Rice University, Houston, Texas 77005, USA
[*] Corresponding author. Prof. Lane W. Martin. Email: lwmartin@rice.edu

## 1 Additional X-ray Structural Characterization

### 1.1 Reciprocal Space Mapping of the $BaTiO_3$ Thickness Series

To assess epitaxy and strain state across the $BaTiO_3$ thickness series, reciprocal space maps (RSMs) were collected about the $GdScO_3$ 332-diffraction condition for the $SrRuO_3/BaTiO_3$ heterostructures with $BaTiO_3$ thicknesses of 25, 50, and 100 nm [Figure S1]. In all cases, the $BaTiO_3$ and $SrRuO_3$ film reflections are aligned with the substrate in-plane, indicating coherent epitaxial growth across the series. The 25- and 50-nm-thick films remain fully coherently strained, while the 100-nm-thick film exhibits the initial onset of relaxation, manifested as a modest broadening and shift of the $BaTiO_3$ reflection, while remaining largely clamped in-plane. This is also consistent with the slight deviation in the XLD data in the main text for this thickness.

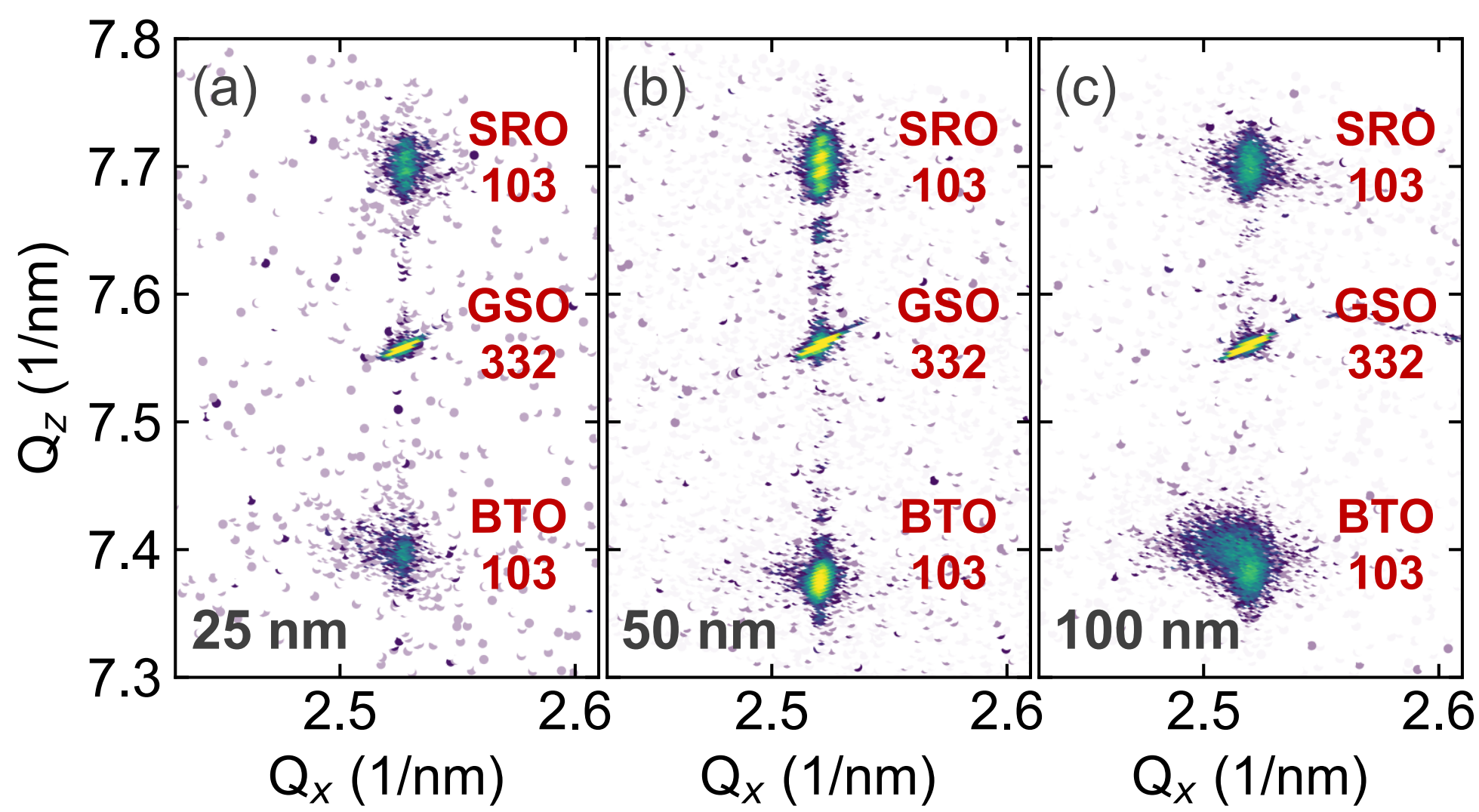


Figure S1: Reciprocal space maps around the $GdScO_3$ 332-diffraction condition for $SrRuO_3/BaTiO_3$ heterostructures with $BaTiO_3$ thicknesses of (a) 25 nm, (b) 50 nm, and (c) 100 nm. In all cases, the $BaTiO_3$ and $SrRuO_3$ reflections are aligned with the substrate in-plane, confirming coherent epitaxy. The 100 nm film shows the initial onset of relaxation while remaining largely clamped in-plane.

### 1.2 Structural Characterization of $SrTiO_3$ Buffer-Layer Heterostructures

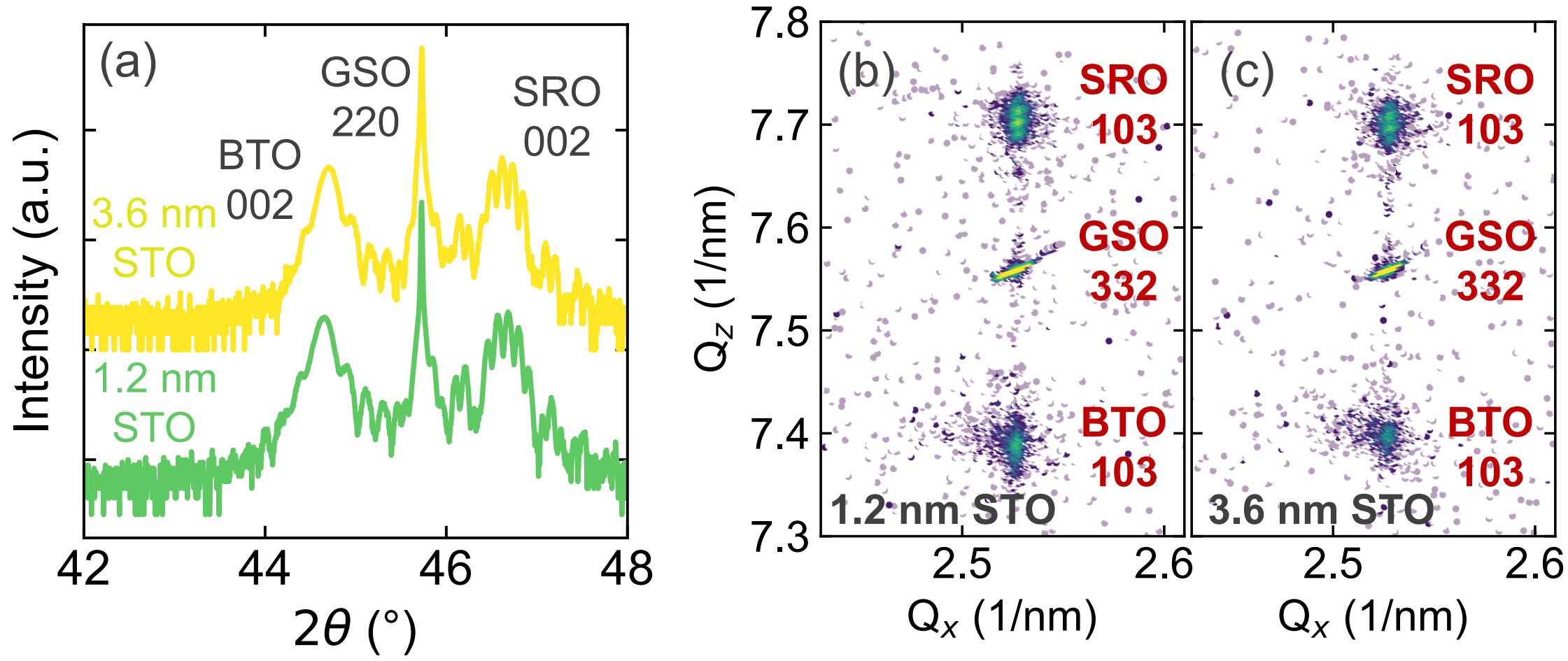


Figure S2: Structural characterization of $SrRuO_3/SrTiO_3/BaTiO_3/SrTiO_3/SrRuO_3$ heterostructures. (a) Symmetric $\omega$-$2\theta$ scans for samples with 1.2 nm and 3.6 nm $SrTiO_3$ buffer layers, showing well-defined $BaTiO_3$, $SrRuO_3$, and substrate reflections. (b,c) Reciprocal space maps around the $GdScO_3$ 332-diffraction condition for heterostructures with 1.2 nm and 3.6 nm $SrTiO_3$ buffer layers, respectively. In both cases, the film reflections remain aligned with the substrate in-plane, confirming coherent epitaxy in the presence of the dielectric buffer layers.

X-ray diffraction and reciprocal space mapping were performed on heterostructures incorporating $SrTiO_3$ buffer layers to assess epitaxy and strain state in the presence of the dielectric interfaces [Figure S2]. Symmetric $\omega$-$2\theta$ scans show well-defined $BaTiO_3$, $SrRuO_3$, and $GdScO_3$ reflections for all buffer-layer thicknesses, with no evidence of secondary phases or degradation of crystallinity.

Reciprocal space maps collected about the $GdScO_3$ 332-diffraction condition show that the $BaTiO_3$ and $SrRuO_3$ reflections remain aligned with the substrate in-plane for both 1.2 nm and 3.6 nm $SrTiO_3$ buffer layers, indicating that the heterostructures remain coherently strained. No additional relaxation or loss of epitaxy is observed upon introduction of the dielectric layers. These measurements confirm that the incorporation of $SrTiO_3$ buffers modifies the electrostatic boundary conditions without introducing structural relaxation or crystallographic disorder.

## 2 X-ray Absorption Spectroscopy and Linear Dichroism of the Polar Distortion

### 2.1 Origin of the Titanium $L$-Edge Dichroism in Polar $BaTiO_3$

The ferroelectric polarization of $BaTiO_3$ originates in the displacement of the titanium ion from the center of its surrounding oxygen octahedron [1]. This off-centering lowers the local site symmetry from cubic to tetragonal and further lifts the degeneracy of the titanium $3d$ levels: the cubic crystal field splits them into $t_{2g}$ and $e_g$ states, and the tetragonal distortion splits each of these in turn into components derived from in-plane and out-of-plane orbitals. Titanium $L_{2,3}$ absorption probes these levels directly through $2p \rightarrow 3d$ transitions [2].

Linearly polarized X-rays select between these components in the following way: X-rays polarized parallel to the polar $c$ axis couple to the out-of-plane-derived $d_{3z^2-r^2}$ and $d_{xz,yz}$ states, and those polarized perpendicular to $c$ to the in-plane $d_{x^2-y^2}$ and $d_{xy}$ states. The difference between spectra recorded in the two geometries therefore reports the orbital-energy asymmetry produced by the off-centering, and it is the presence of a dichroism at the $t_{2g}$ and $e_g$ features, together with its sign, that constitutes the spectroscopic signature of the polar state [3]. The sign observed throughout this work, with the out-of-plane response lying below the in-plane response at the $e_g$ feature, places $d_{3z^2-r^2}$ below $d_{x^2-y^2}$, as expected for a unit cell elongated along the polar axis.

Because the dichroism reports the distortion of individual unit cells and is insensitive to their relative orientation, a film containing oppositely poled domains is indistinguishable from a uniformly poled one. The measurement therefore tests the polar distortion independently of the domain configuration, reporting its symmetry and its relative magnitude between samples rather than an absolute dipole moment.

### 2.2 Data Reduction

The measurement geometry, the recovery of the out-of-plane response $I_c$, and the construction of the dichroism $\Delta I(E) = I_c(E) - I_{ab}(E)$ are given in the Experimental Section. The treatment applied to the spectra is as follows. Spectra at each polarization were averaged over four successive scans, normalized to the incident photon flux, and interpolated onto a common energy grid without alignment between polarizations, the offset between them being the quantity of interest. A linear pre-edge background was subtracted (450–453 eV at the titanium edge, 515–525 eV at the oxygen edge) and each spectrum normalized to its own post-edge continuum (480–490 eV and 575–590 eV, respectively) before the geometric decomposition was applied. Because every spectrum is normalized to its own edge jump, the dichroism is expressed as a fraction of the absorption edge of the same measurement, so its amplitude does not depend on the overall signal level of the sample from which it was obtained. No smoothing or energy shifting was applied at any stage.

### 2.3 Capped Heterostructures and Removal of the $SrRuO_3$ Contribution

Because the electrostatic boundary conditions of an uncapped film differ from those of a completed capacitor, heterostructures capped with $SrRuO_3$ were measured alongside the uncapped series. The capping layer, at 3 nm, is thin enough for the electron yield to reach the $BaTiO_3$ beneath while still presenting a metallic boundary comparable to that of the device electrodes.

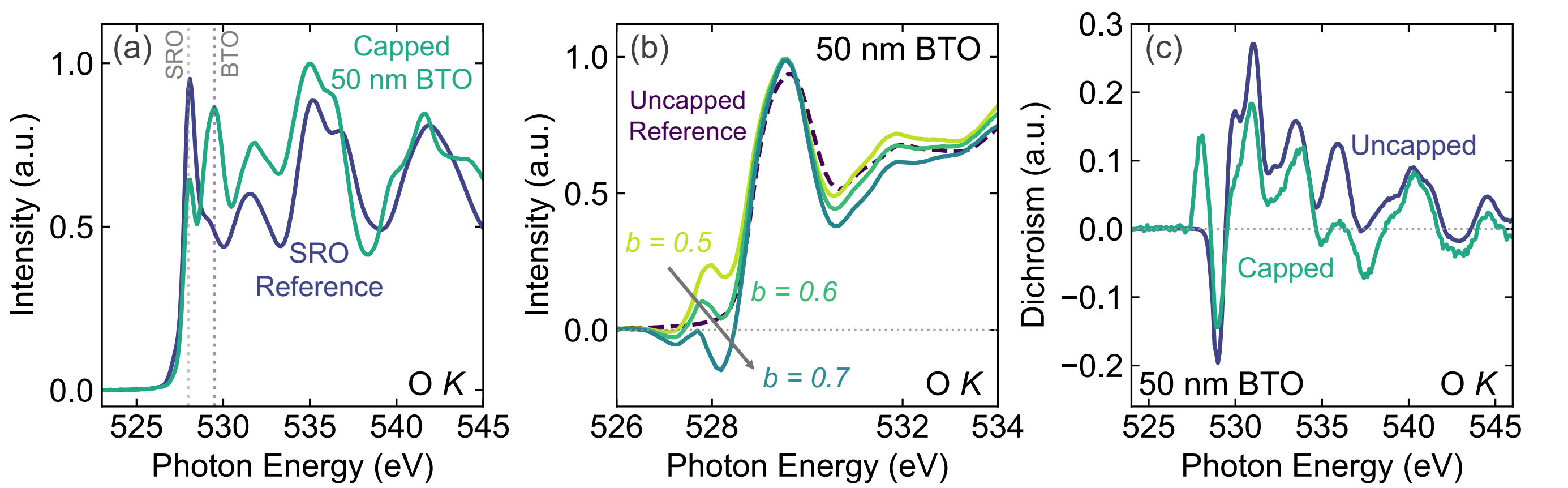


Figure S3: Oxygen $K$-edge spectra and removal of the $SrRuO_3$ contribution. (a) Bare $SrRuO_3$ reference spectrum together with a capped heterostructure, showing the $SrRuO_3$ onset near 528 eV that distinguishes the two contributions. (b) Determination of the $SrRuO_3$ fraction $b$ for a representative capped sample: the reference is scaled and subtracted, and $b$ adjusted until the 528 eV onset is minimized in the remainder. (c) Recovered oxygen-edge dichroism of the capped film alongside its uncapped counterpart. The residual feature near 528 eV arises from interfacial oxygen not represented by the $SrRuO_3$ reference.

The measured signal of these heterostructures contains contributions from both $BaTiO_3$ and $SrRuO_3$, as does that of the 3-nm-thick uncapped film, in which the electron yield reaches the bottom electrode. Across the titanium $L_{2,3}$ edges the $SrRuO_3$ contribution is broad and overlaps the titanium features, while at the oxygen $K$ edge it is spectrally distinct, rising near 528 eV against approximately 529.5 eV for $BaTiO_3$ [Figure S3(a)]. The $SrRuO_3$ fraction is therefore determined at the oxygen edge and applied to the titanium spectra of the same sample. A bare $SrRuO_3$ film grown under identical conditions provides the reference: it was scaled by a fraction $b$, subtracted from the measured spectrum, and $b$ adjusted until the $SrRuO_3$ onset near 528 eV was minimized in the remainder [Figure S3(b)], after which the remainder was re-normalized by $1/(1-b)$ so that the post-edge continuum returns to unity. The $SrRuO_3$ contribution accounts for approximately 55–60% of the measured signal across the capped heterostructures, and approximately 45% for the 3-nm-thick uncapped film, where what is removed is the underlying electrode seen through the film rather than a capping layer above it.

A residual feature survives near 528 eV in the capped oxygen dichroism [Figure S3(c)]: oxygen at the $BaTiO_3$–$SrRuO_3$ interface is chemically distinct from oxygen in either constituent, and is therefore not fully accounted for by a reference measured on $SrRuO_3$ alone. That region is not interpreted quantitatively.

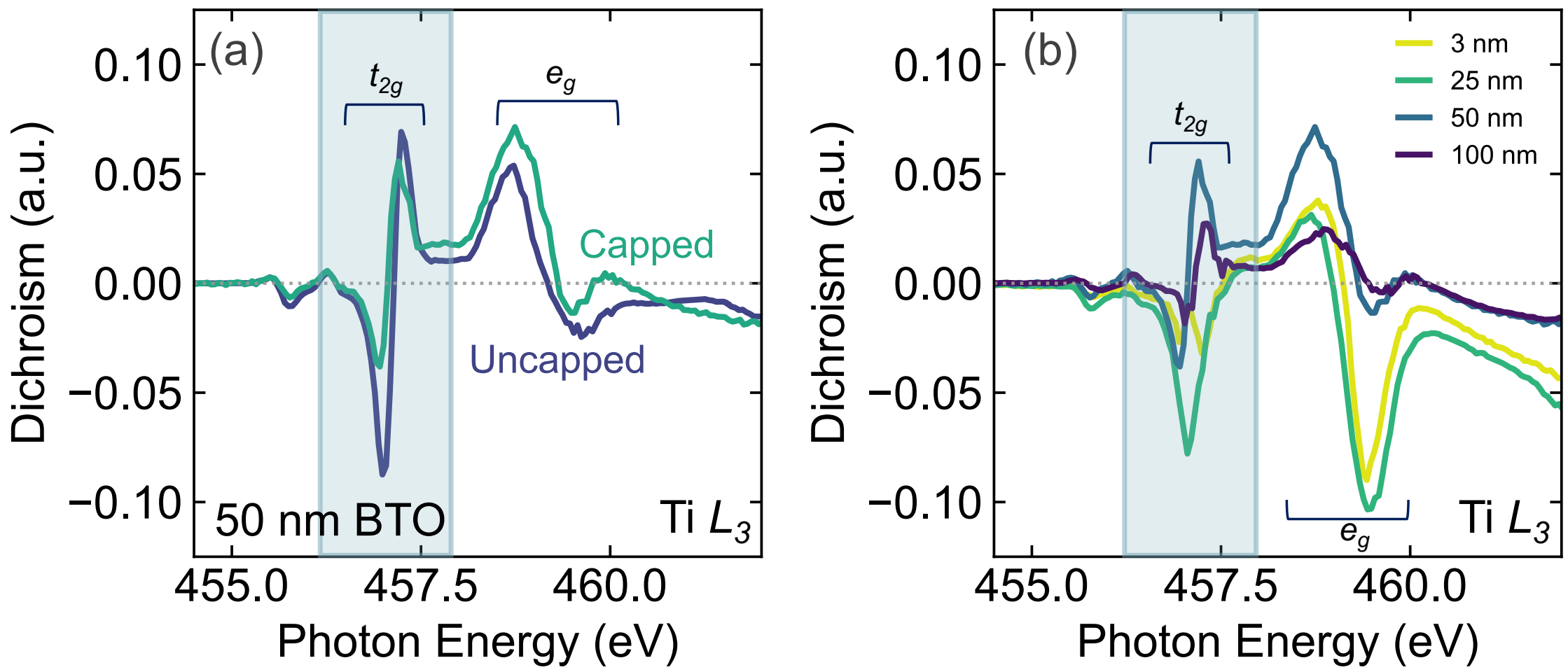


Figure S4: Comparison of capped and uncapped heterostructures at the titanium $L_{2,3}$ edges. (a) Titanium-edge dichroism of a capped and an uncapped 50 nm $BaTiO_3$ film. (b) Comparison across the full thickness series of capped samples. The dichroism is qualitatively similar near the titanium $L_3$ $t_{2g}$ under capping at every thickness, however, the interface-dominated nature of the capped measurement increases variability across the thickness range measured.

With the $SrRuO_3$ contribution removed, the capped heterostructures show dichroism at the $t_{2g}$ and $e_g$ features qualitatively similar to that of the uncapped films at every thickness [Figure S4]. The amplitudes of the two geometries are not directly comparable, for the same reason the oxygen residual persists: the electron yield is weighted toward the top of the film ($\approx$ 5 nm) [4], so in a capped heterostructure the detected titanium lies predominantly within the region participating in the $BaTiO_3$–$SrRuO_3$ interface, whereas in an uncapped film the same weighting falls on $BaTiO_3$ terminated by a free surface. Within the uncapped series, where no such distinction arises, the amplitudes are directly comparable, and it is the uncapped measurements – the more demanding electrostatic case, with the polarization least screened and the depolarizing field correspondingly largest – that are reported in the main text.

# 3 Extraction of Transient Depolarization Fields

## 3.1 Polarization Extraction from PUND Measurements

The transient remanent polarization $P(t)$ is extracted from pulsed-switching (PUND) measurements. At each delay time, the switched polarization is obtained by subtracting the non-switching response from the switching transient and integrating the resulting current, normalized by device area. Each device was cycled repeatedly in both polarization states, and the mean $P(t)$ and standard deviation $\sigma_P$ were determined from measurements across devices within each sample.

## 3.2 Nucleation-and-Growth Model Inversion

The transient depolarization field $E_d(t)$ is extracted by inverting the nucleation-and-growth model (Equation 1 of the main text). During polarization relaxation no external field is applied, so the field driving domain-wall motion is purely the internally generated depolarization field, $E_{FE}(t) = E_d(t)$. The governing equations are:

$$\frac{dP(t)}{dt} = 2\pi n u(t) P_{sw} \int_0^t u(t')\, dt', \tag{S1}$$

$$u(t) = u_0 \exp[-E_a/E_d(t)]\,. \tag{S2}$$

The integral $\int_0^t u(t')\, dt'$ is the domain radius $r(t)$ – the radial extent of growth from a nucleation site at time $t$. Defining $r(t) = \int_0^t u_0 \exp[-E_a/E_d(t')]\, dt'$ explicitly, Equation S1 can be rearranged to give an explicit expression

for $E_d$ at each time step:

$$E_d(t) = \frac{-E_a}{\ln[(dP/dt) \,/\, (2\pi n P_{sw} u_0 r(t))]}. \tag{S3}$$

Because $u(t)$ is monotonically increasing in $E_d$, this inversion is unique at each step. The solution proceeds by stepping forward in time: at each $t_i$, the domain radius $r(t_i)$ is updated from all prior steps, and $E_d(t_i)$ is obtained directly from Equation S3. The time derivative $dP/dt$ required at each step is evaluated numerically from the measured $P(t)$ using centered finite differences.

### 3.3 Uncertainty Propagation

The primary source of uncertainty considered for all derived quantities is $\sigma_P$. The measurement uncertainty on $dP/dt$ propagates as

$$\sigma_{dP/dt}(t_i) = \frac{\sqrt{2}\,\sigma_P}{t_{i+1} - t_{i-1}}. \tag{S4}$$

Differentiating Equation S3 with respect to $dP/dt$, treating $r(t)$ as fixed at each step, gives the propagated uncertainty on the depolarization field:

$$\sigma_{E_d} = \frac{E_d^2}{E_a} \cdot \frac{\sigma_{dP/dt}}{dP/dt}. \tag{S5}$$

As the system approaches equilibrium, $dP/dt \to 0$ and $\sigma_{E_d}$ diverges, producing the growth in error bars at long delay times in Figure 2(c) of the main text.

The interfacial screening capacitance is extracted from the $(P,\ E_d)$ pairs via $C_{scn} = 2PA/(E_d\, t_{FE})$. Propagating $\sigma_P$ and $\sigma_{E_d}$ through this expression gives

$$\sigma_{C_{scn}} = C_{scn}\sqrt{\left(\frac{\sigma_P}{P}\right)^2 + \left(\frac{\sigma_{E_d}}{E_d}\right)^2}. \tag{S6}$$

## 4 Electrostatic and Phase-Field Modeling

### 4.1 Analysis of Interfacial Dielectrics

To validate the series-capacitance model and demonstrate that the dielectric-buffer layers modify the polarization stability through electrostatic mechanisms, the effective interfacial capacitance was extracted from the experimental depolarization-field data. Using the measured pairs of $(P, E_d)$ from PUND measurements and the series-capacitance relationship

$$E_d = \frac{2P}{C_{ser} \cdot t_{FE}}, \tag{S7}$$

where the total interfacial series capacitance $C_{ser}$ was back-calculated for each buffer-layer configuration. The extracted capacitance follows the expected series combination [Fig. S5(b)]:

$$\frac{1}{C_{ser}} = \frac{2}{C_{scn}} + \frac{2}{C_{STO}}, \tag{S8}$$

where $C_{scn}$ is the $SrRuO_3$ screening capacitance (constant across all samples) and $C_{STO}$ is the capacitance of the $SrTiO_3$ buffer layer.

As the dielectric thickness increases from 0 to 3.6 nm, $C_{ser}$ decreases systematically, reducing the effective screening of the polarization and thereby increasing the magnitude of the depolarization field at a given polarization state [Figure S5]. This quantitative agreement confirms that dielectric buffering tunes the polarization stability through the same electrostatic mechanism as thickness scaling – by modifying the series capacitance at the ferroelectric-electrode interface.

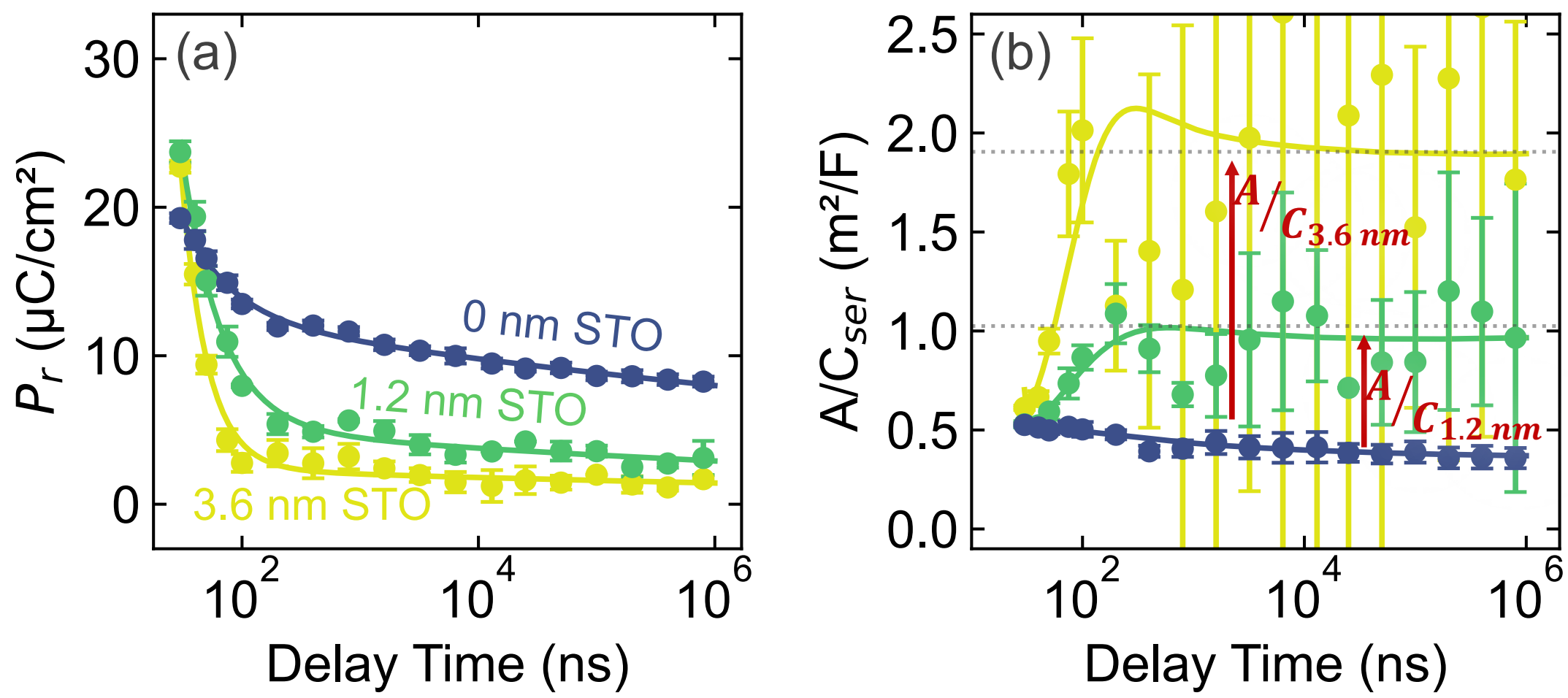


Figure S5: (a) Transient remanent polarization for 50 nm $BaTiO_3$ with varying $SrTiO_3$ buffer layer thickness (0, 1.2, and 3.6 nm), showing systematic reduction in $P_r$ with increasing dielectric thickness. (b) Extracted effective series-capacitance density ($A/C_{ser}$) versus delay time for the same devices. All samples converge to equilibrium values consistent with series-capacitance predictions, validating that the series combination of electrode screening ($C_{scn}$) and dielectric buffer ($C_{STO}$) governs the capacitive response across all delay timescales.

## 4.2 Phase-Field Validation: Ferroelectric Energy Landscape Remains Unchanged

While dielectric-buffer layers produce dramatic changes in macroscopic polarization-electric field hysteresis loop shape [Figure S6(a)], the underlying ferroelectric free-energy landscape of $BaTiO_3$ remains unaffected. Decomposition of the total free energy into its constituent terms [Figure S6(b)] shows that all energy components behave identically across all dielectric configurations when plotted as a function of polarization state. Thus, with respect to polarization, the ferroelectric responds in the same manner regardless of dielectric thickness.

This energy analysis reveals the physical origin of the loop broadening: thicker dielectric layers do not weaken the ferroelectric or make switching easier. Instead, they increase the internal depolarization field and redistribute the applied voltage away from the ferroelectric layer. Under a given applied field, the ferroelectric reaches lower polarization values because more voltage drops across the dielectric buffer. The broader switching transition thus reflects the system entering a regime where depolarization-driven domain nucleation initiates at lower applied fields, but the larger opposing field slows propagation. Once the electrostatic boundary conditions are accounted for through the effective field $E_{FE}$, all devices collapse onto the same universal curve, demonstrating that the intrinsic switching physics of $BaTiO_3$ is preserved.

## 4.3 Phase-field Calibration: Defect-mediated Nucleation and Growth

Phase-field simulations were used to model domain-mediated switching and to assess how electrostatic boundary conditions influence polarization stability. The model employs a time-dependent Ginzburg-Landau formulation for $BaTiO_3$ with a single out-of-plane polarization component (consistent with the measured $c$-axis orientation) and self-consistent electrostatics implemented via finite extrapolation-length boundary conditions [5].

In an idealized defect-free simulation with fully homogeneous material parameters, polarization reversal proceeds through a spatially uniform reduction of the polarization magnitude rather than by nucleation and lateral growth of domains [Figure S7(b)]. Such homogeneous reversal is a known artifact of perfectly symmetric phase-field models lacking heterogeneity [6], and it is not representative of experimentally observed switching pathways in high-quality ferroelectric thin films [7, 8, 9].

To recover a physically realistic nucleation-and-growth pathway while preserving a robust local polarization magnitude during switching, a dilute population of heterogeneous defect sites was introduced. Two classes were used: nucleation sites that locally reduce the reversal barrier and pinning sites that impede domain-wall motion through a localized increase of the Landau coefficient. With these perturbations, reversal occurs through discrete nucleation events followed by domain-wall propagation and coalescence [Figure S7(b)], providing a calibrated baseline for subsequent simulations of depolarization-driven relaxation and interfacial-capacitance effects.

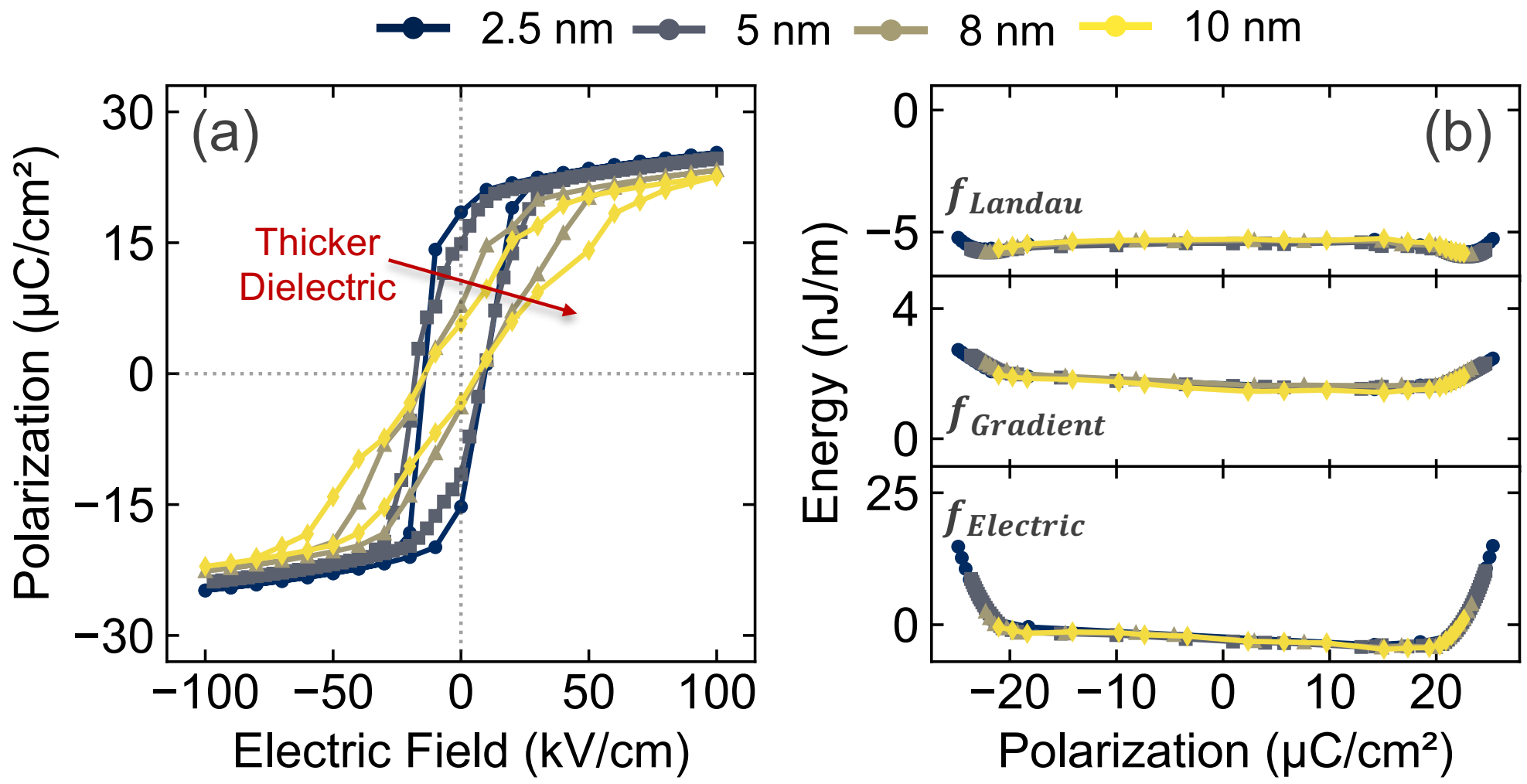


Figure S6: (a) Polarization-electric field hysteresis loops from phase-field simulations of 50 nm $BaTiO_3$ with dielectric layers of varying thickness (2.5-10 nm) at the interfaces, showing the characteristic broadening of switching transitions. (b) Decomposition of the total free energy into Landau, gradient, and electrostatic contributions as a function of polarization. The Landau and gradient energies remain essentially unchanged across all dielectric thicknesses, confirming that the intrinsic switching landscape of $BaTiO_3$ is preserved. Differences in loop shape arise purely from voltage redistribution and electrostatic boundary conditions, not from modifications to the ferroelectric energy landscape.

## 5 Dielectric Characterization

Dielectric measurements on the $BaTiO_3$ thickness series and $SrTiO_3$ dielectric-buffer heterostructures complement the depolarization analyses and provide capacitance values for use in the electrostatic modeling. Figure S8 shows that thinner $BaTiO_3$ films exhibit higher zero-field permittivity and progressively broader dielectric peaks, with the 25-nm-thick films showing the broadest response. At high applied fields, permittivity converges across all thicknesses, indicating that varying thickness primarily affects the field range over which the dielectric response evolves, but leaves the linear material response unchanged.

### 5.1 Effect of $SrTiO_3$ Buffer Layers

Introducing $SrTiO_3$ buffer layers produces similar behavior: a 1.2 nm layer modestly broadens the permittivity peak, while 3.6 nm further broadens it and reduces peak permittivity [Figure S9]. Again, high-field permittivity remains largely unchanged, confirming that dielectric buffering redistributes the applied voltage without necessarily altering the intrinsic ferroelectric response.

These trends parallel the polarization hysteresis measurements and support the interpretation that both thickness scaling and dielectric buffering modify electrostatic boundary conditions, broadening the field range over which polarization evolves. The permittivity values extracted from these measurements serve as baseline inputs for electrostatic modeling of depolarization behavior and for subsequent corrections applied to the polarization–electric field data, providing essential material parameters for the analyses presented in the main text.

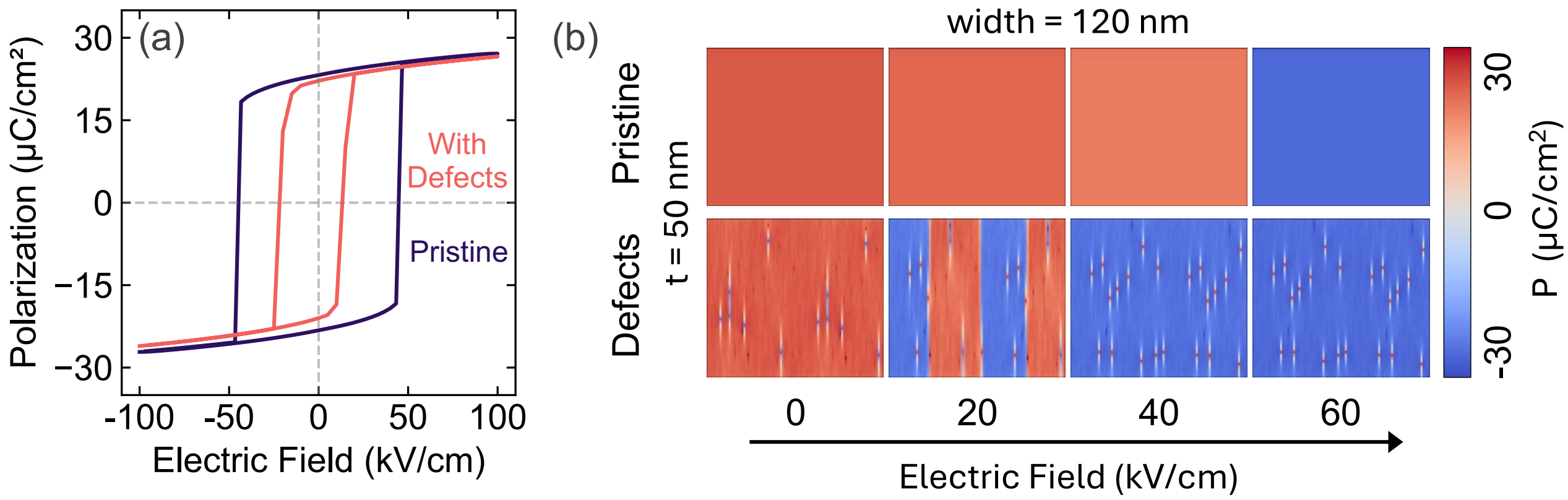


Figure S7: Phase-field calibration of switching pathways in $BaTiO_3$. (a) Polarization-electric field hysteresis loops from simulations without defects and with a dilute population of heterogeneous defect sites. (b) Corresponding domain maps illustrating spatially homogeneous polarization reversal in the defect-free case and nucleation-and-growth-mediated switching when heterogeneous sites are included.

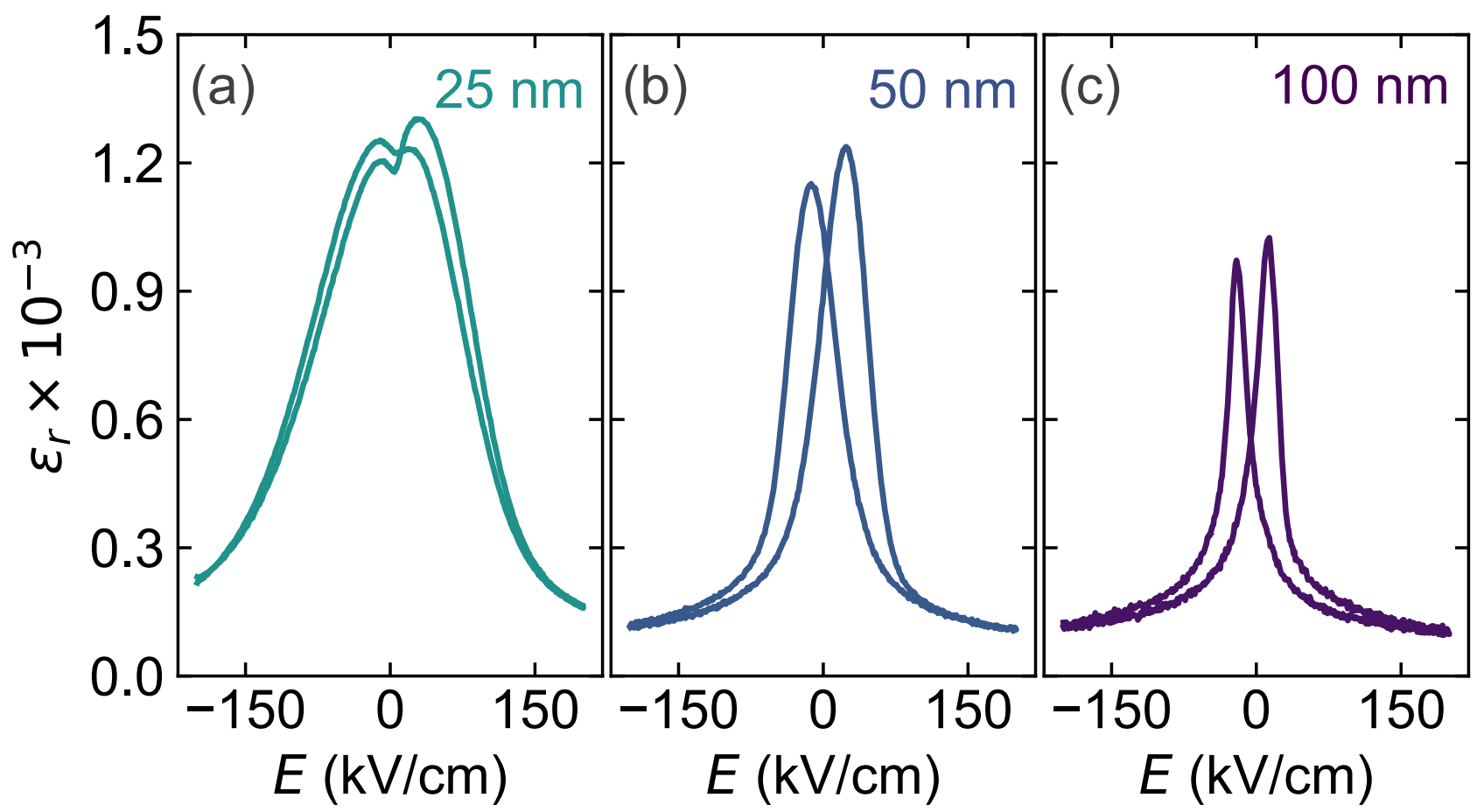


Figure S8: Electric-field dependence of the relative permittivity $\varepsilon_r$ for $BaTiO_3$ films with thicknesses of (a) 25 nm, (b) 50 nm, and (c) 100 nm. Thinner films exhibit higher zero-field permittivity and broader dielectric peaks, while the high-field dielectric response remains similar across all thicknesses.

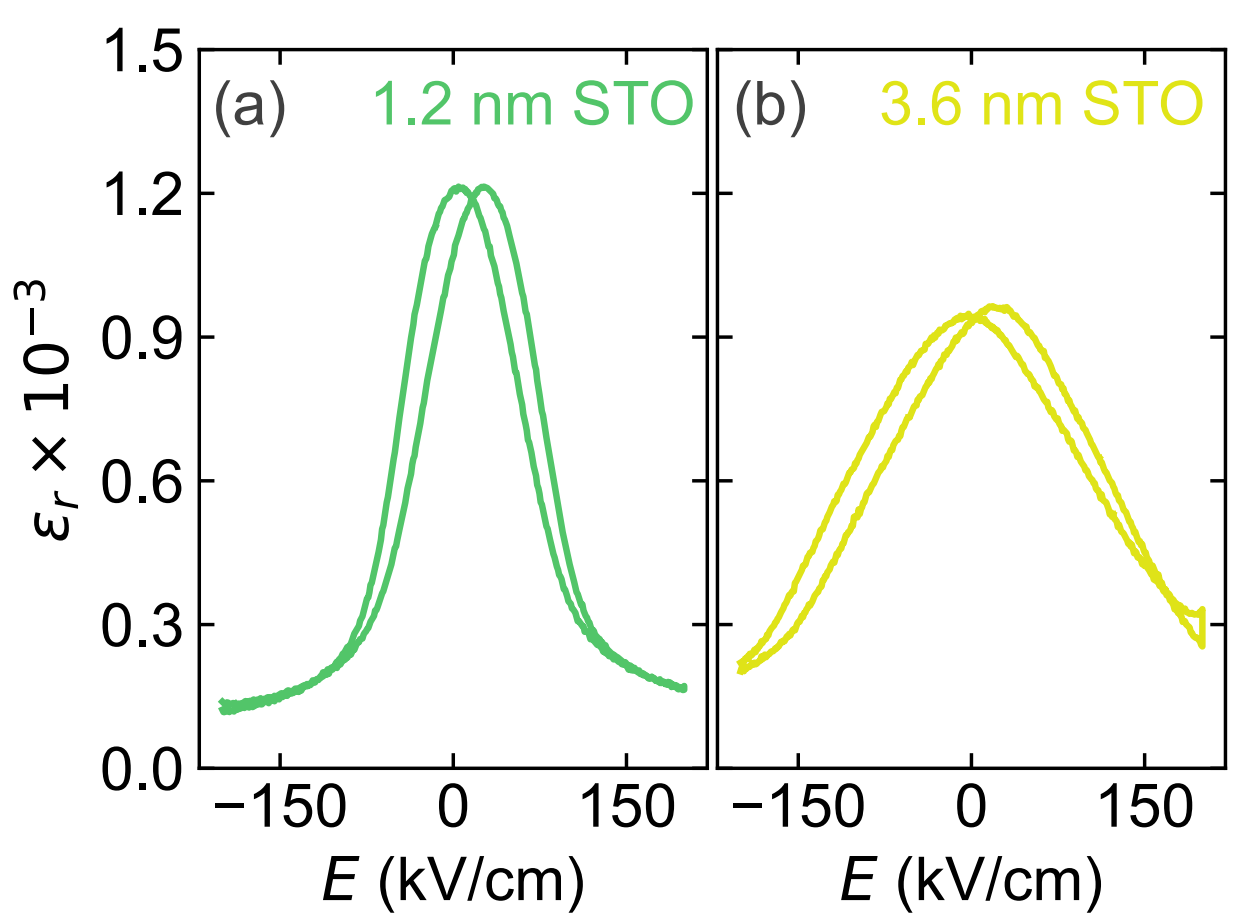


Figure S9: Electric-field dependence of the relative permittivity $\varepsilon_r$ for 50 nm $BaTiO_3$ films with (a) 1.2 nm, and (b) 3.6 nm $SrTiO_3$ buffer layers. Increasing dielectric thickness broadens the permittivity peak and reduces its maximum value, while the high-field dielectric response remains largely unchanged.

## References


[1] R. E. Cohen, *Nature* **1992**, *358*, 6382 136.

[2] de Groot FM, J. C. Fuggle, B. T. Thole, G. A. Sawatzky, *Phys. Rev. B Condens. Matter* **1990**, *42*, 9 5459.

[3] Y. Song, X. Liu, F. Wen, M. Kareev, R. Zhang, Y. Pei, J. Bi, P. Shafer, A. T. N'Diaye, E. Arenholz, S. Y. Park, Y. Cao, J. Chakhalian, *Phys. Rev. Mater.* **2020**, *4*, 2 024413.

[4] M. Abbate, J. B. Goedkoop, F. M. F. de Groot, M. Grioni, J. C. Fuggle, S. Hofmann, H. Petersen, M. Sacchi, *Surf. Interface Anal.* **1992**, *18*, 1 65.

[5] P. Kumar, A. Nonaka, R. Jambunathan, G. Pahwa, S. Salahuddin, Z. Yao, *Comput. Phys. Commun.* **2023**, *290*, 108757 108757.

[6] L.-Q. Chen, *Annu. Rev. Mater. Res.* **2002**, *32*, 1 113.

[7] W. J. Merz, *Phys. Rev.* **1954**, *95*, 3 690.

[8] J. Y. Jo, D. J. Kim, Y. S. Kim, S.-B. Choe, T. K. Song, J.-G. Yoon, T. W. Noh, *Phys. Rev. Lett.* **2006**, *97*, 24 247602.

[9] J. F. Scott, *Ferroelectrics* **2016**, *503*, 1 117.
